# Antisense transcription can induce expression memory via stable promoter repression

**DOI:** 10.1186/s13059-025-03875-1

**Published:** 2025-12-20

**Authors:** Verena Mutzel, Till Schwämmle, Svearike Oeverdieck, Lucija Librenjak, Benedikt Boesen, Melissa Bothe, Rutger A. F. Gjaltema, Ilona Dunkel, Gemma Noviello, Edda G. Schulz

**Affiliations:** Systems Epigenetics, Otto Warburg Laboratories, Max Planck Institute for Molecular Genetics, 14195 Berlin, Germany

**Keywords:** Antisense transcription, Mathematical modelling, Synthetic genomics, Systems biology, Embryonic stem cells, Transcriptional regulation, Epigenetics

## Abstract

**Background:**

The capacity of cells to retain a memory of previous signals enables acquisition of unique fates and adaptation to their environment. The underlying gene expression memory can arise from mutual repression of two genes, forming a toggle switch. Mutual repression can occur at antisense loci, where convergent genes repress each other in *cis*. The conditions for generating expression memory via antisense transcription remain poorly understood. To address this question, we combine mathematical modeling, genomics and a synthetic biology approach.

**Results:**

Simulations demonstrate stable memory emergence when both genes in an antisense pair transcribe via the convergent promoter and induce a stable repressive chromatin state. Genome-wide analysis of nascent transcription supports antisense-mediated promoter repression, since promoter-overlapping antisense gene pairs exhibit mutually exclusive expression. Through constructing a synthetic antisense locus in mESCs, we demonstrate that antisense transcription can induce stable repression, a key prerequisite for memory. Repression stability increases during mESC differentiation, highlighting cell type-specific epigenetic memory.

**Conclusions:**

Our work establishes a quantitative framework which predicts that antisense-mediated cis-memory can arise within physiologically relevant conditions, and shows that a biological phenomenon with kinetics in the range of weeks can emerge from the interplay of multiple faster molecular processes. This framework, combined with our experimental findings, demonstrates how antisense transcription can encode stable gene expression states. Our discovery that stem cells adjust their memory capacity during differentiation may clarify mechanisms underlying stemness maintenance.

**Supplementary Information:**

The online version contains supplementary material available at 10.1186/s13059-025-03875-1.

## Background

Multicellular life requires the ability to memorize past signals. During embryonic development, cells must progressively commit to distinct cell lineages and stably remember their identity long after the commitment-inducing signals are gone. Also adaptation to environmental conditions frequently requires a memory of transient cues. The underlying gene expression memory can be established at the level of transcription factor networks in *trans* or at an individual allele in *cis*. *Trans*-memory uniformly affects all gene copies in a cell, whereas *cis*-memory enables individual alleles to retain distinct transcriptional states. Binary cell-fate decisions based on *trans*-memory are often achieved through cross-inhibition between two fate-determining transcription factors, a network motif called a toggle switch [1, 2]. *Cis*-memory can be generated by self-reinforcing chromatin modifications, for example established through the Polycomb system [3]. In addition, many *cis*-memory genes are regulated by antisense transcription (see below). Such an overlapping, convergent orientation of two genes could result in mutual repression, and potentially form a *cis*-acting toggle switch to generate *cis*-memory. Due to the switch-like nature, only one of the two genes would be efficiently transcribed at a time while the other is repressed. The term “mutual repression” thus refers to the ability of a gene pair to repress each other, however at any given time, repression is only exerted by the transcriptionally active gene.

Genomic imprinting is a prime example for *cis*-memory, where parental origin determines the allelic expression state. Antisense transcripts contribute to maintaining allele-specific gene repression at many imprinted loci [4]. Also the *Xist* locus, which governs X-chromosome inactivation, is controlled by an antisense transcript in mice, called Tsix [5]. After Xist-driven inactivation of one randomly chosen X chromosome, Xist continues to be expressed from the inactive X throughout life, thus maintaining a memory of the initial random decision. Using mathematical modeling, we have previously shown that antisense transcription at the *Xist/Tsix* locus could help to lock in alternative expression states at the two alleles [6]. More recently, antisense transcription has been described as a general hallmark of genes exhibiting random monoallelic expression [7], where it might have a functional role in stabilizing allelic expression states.

Given the pervasive transcription of the genome, >30% of coding genes exhibit antisense transcription [8], though the term encompasses various phenomena. Many mammalian promoters show both divergent and convergent antisense transcription, originating slightly upstream and downstream of the transcription start site (TSS), respectively, producing short (< 500 bp) unstable transcripts [9–12]. Contrarily, regulatory antisense RNAs at imprinted gene clusters and Xist are longer (>10 kb), typically spliced transcripts with their own promoter [13], enabling responsiveness to stimuli independently from the sense gene.

A variety of mechanisms have been described to underlie antisense-mediated gene repression [13]. Besides sequence-specific modes of regulation, repression can occur through transcriptional interference, where transcription itself mediates the repression. Collisions of two RNA polymerases transcribing in opposite directions can result in removal of one or both polymerases from the DNA [14]. The frequency of collisions largely depends on the length of the overlapping region and the transcription rates, but their importance for regulation in vivo remains unknown. More potent repression by contrast might occur when the antisense gene is transcribed through the sense promoter. The antisense polymerase can interfere with the formation of the pre-initiation complex, leading to so-called sitting-duck-interference [15], and can alter chromatin modifications at the promoter. Transcriptional elongation promotes the establishment of repressive marks, such as H3K36me3, which in turn recruits DNA methylation [16–20]. This mechanism is thought to suppress transcription initiation within actively transcribed genes, but also has a functional role in antisense-mediated repression, e.g. at the *Xist* promoter [21]. Chromatin-mediated repression can, in principle, function at lower transcription rates compared to sitting-duck-interference or polymerase collisions, since the repressed state can be maintained after the polymerase has passed.

**Fig. 1:**
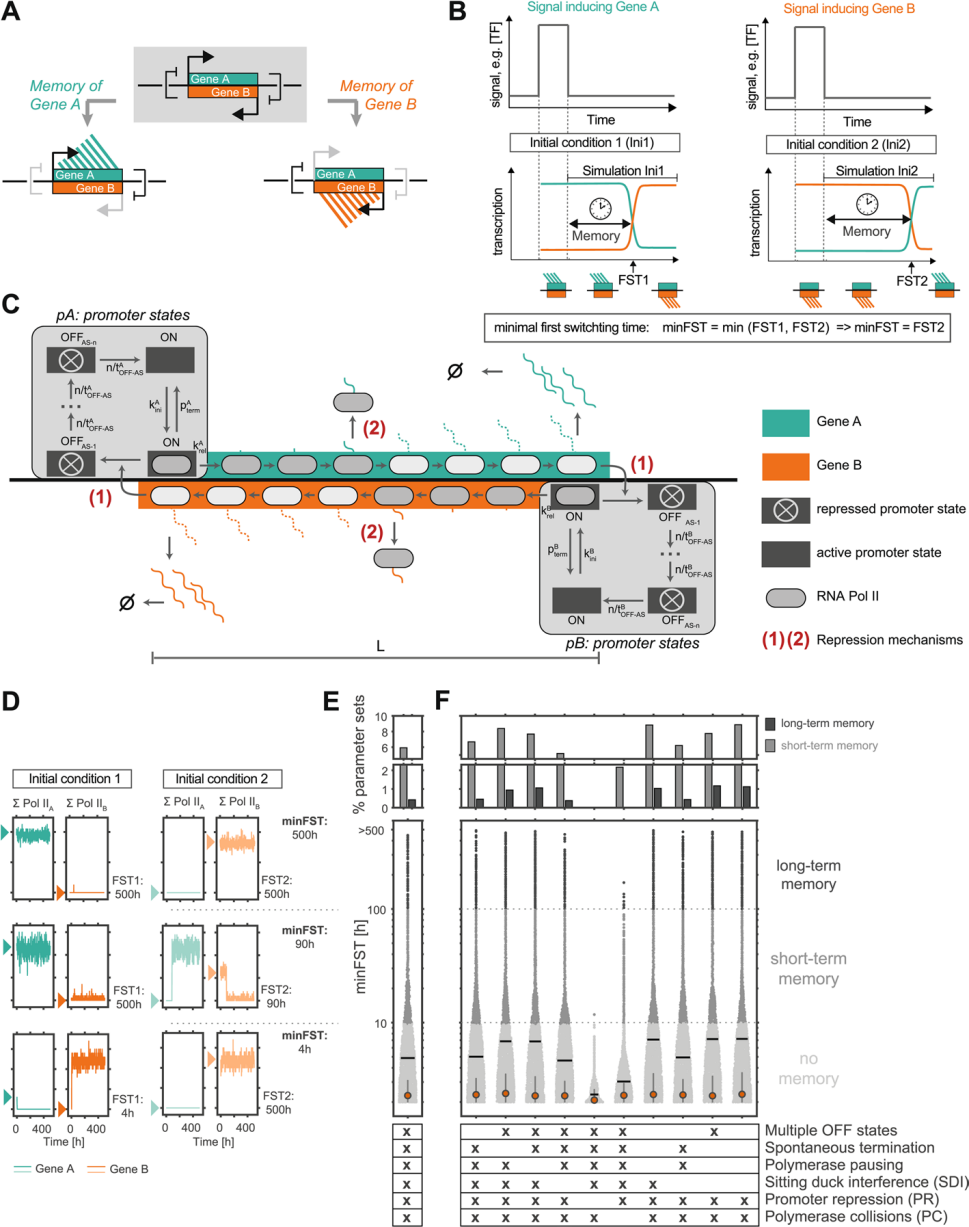
Mathematical model predicts that convergent promoters can memorize alternative expression states. **A** Scheme of an antisense locus with two convergently oriented genes that mutually repress each other to form a toggle switch, that can maintain a memory of two alternative expression states. **B** Schematic representation of allelic expression memory at an antisense locus. Left: In initial condition (Ini) 1 a transient transcription factor (TF) signal has induced Gene A leading to repression of Gene B. Right: In Ini2 a transient signal has induced Gene B leading to repression of Gene A. The simulations D-F start once the transient signal is gone and measure the transcriptional memory, defined as the time that passes until the locus’ transcription state switches (FST1, FST2). minFST denotes the minimum of the two FSTs. **C** Scheme of the mathematical model of antisense transcription, where two genes A and B overlap at a genomic segment of length L. Pol II complexes can initiate at both convergent promoters, when these are in the ON state, with distinct initiation rates (k^A^_ini_, k^B^_ini_), and can then either be released into elongation (k^A^_rel_, k^B^_rel_), or spontaneously terminate transcription shortly downstream of the promoter (p^A^_term_, p^B^_term_). Elongating Pol II complexes move along the gene in convergent orientation. Mutual repression occurs through the act of transcription on the level of (1) promoter silencing by antisense transcription (ON-to-OFF transition induced by antisense Pol II at the promoter), and (2) transcriptional interference between two antisense Pol II complexes that occupy the same DNA segment, resulting in dislodgement of one Pol II from the DNA. **D** Representative Gillespie simulation of transcription for an individual allele (sum of Pol II complexes on the gene, ∑Pol II) on gene A and B, as indicated on top. Three example parameter sets with no (bottom), short-term (middle), and long-term (top) expression memory are shown. Arrowheads indicate initial transcription state. **E** The minFST, as indicated in B, was averaged across 100 simulated alleles for 35,000 parameter sets, which were then classified into displaying no (light gray, minFST < 10 h), short-term (gray, 10 h < minFST < 100 h), and long-term (dark gray, minFST > 100 h) expression memory for the model shown in C. Top: Percentage of parameter sets displaying short-term (gray) and long-term (dark gray) expression memory. Bottom: Distribution of minFST. **F** Same as in E, but for a series of reduced models, where the process of transcription was simplified or mechanisms of repression between the two antisense strands were removed, as indicated. Each reduced model was simulated with 35,000 parameter sets. In E-F, small dots represent individual parameter sets, the red dots show the median, the horizontal black bars the mean and the whiskers the interquartile range

Since antisense gene pairs can interfere with each other’s transcription through various transcription-mediated mechanisms, mutual repression could in principle establish a toggle switch at antisense loci resulting in mutually exclusive expression. Transcription of each gene, once established, would be self-reinforced through inhibition of its repressive antisense partner, thus stabilizing alternative expression states (Fig. 1A). It remains, however, largely unknown under which conditions antisense loci can maintain expression memory and whether it is facilitated by specific locus architectures or repression mechanisms. Furthermore, it is unclear whether and how a long-term phenomenon like transcriptional memory over several days or weeks can emerge from molecular processes that happen on much faster timescales.

Here, we combine mathematical modeling, genome-wide profiling and a synthetic-biology approach to elucidate the conditions under which antisense transcription can memorize a past signal. Through a series of simulations we show that antisense transcription could maintain expression memory up to several weeks, if both genes transcribe through each other’s promoter at a similar high rate and induce stable promoter repression. Using a time course data set of differentiating mouse embryonic stem cells (mESCs), measuring genome-wide nascent transcription we find that such antisense pairs with promoter overlap show indeed mutually exclusive expression, which becomes more pronounced during differentiation. We then build a synthetic antisense locus to demonstrate that antisense transcription can induce stable promoter repression to retain short-term memory (~ 1 day) in undifferentiated mESCs, which is stabilized during differentiation (> 4 days). Overall our interdisciplinary approach shows that antisense loci can memorize alternative expression states and that this property depends on the locus architecture, transcription rates and the global cellular state.

## Results

### Mathematical model predicts that expression memory can arise at antisense loci

We set out to investigate whether and to what extent mutual repression of two genes by convergent transcription can stably maintain alternative expression states (Fig. 1A + B). Specifically, we analyzed whether a transient signal (e.g. a change in transcription factor abundance or enhancer-promoter interaction frequency), inducing expression of one of the transcripts, could lead to a persistent change in the transcription state of the locus (*cis*-encoded memory).

We developed a mechanistic mathematical model of a generic antisense locus describing two convergent transcripts, Gene A and Gene B (Fig. 1C; Table 1). The model accounts for transcription initiation and promoter-proximal pausing (incl. pre-initiation complex assembly - k_ini_, release from promoter-proximal pausing - k_rel_ and promoter-proximal termination - p_term_), RNA polymerase II (Pol II) elongation, and RNA degradation of the antisense pair. Time-course experiments have suggested that activation of mammalian genes is a multi-step process [27–29]. To account for this observation, we modeled promoter transitions as an irreversible cycle of one transcriptionally active state (ON) and n inactive states (OFF) (Fig. 1C) [25, 30].

**Table 1:** Model parameters. Parameter ranges tested for the different model versions used in Fig. 1–3. The parameters were sampled either from a logarithmic (log) or from a linear (lin) distribution, as indicated. Physiologic parameter ranges were derived from previous experimental estimates: k_ini_,[22], k_rel_ [22, 23], p_term_ [23, 24], t_OFF−AS_ [22, 25], n [25], k_elong_ [26]. For the bursting model, 160 parameter sets displaying long-term memory (>400 h) in the absence of bursting were each combined with 200 log-sampled burst parameters

		Full Model (Fig. 1)	3’ Overlap (Fig. 2)	intragenic overlap (Fig. 2)	promoter overlap (Fig. 2)	Bursting Model (Fig. 3)
Pol II initiation rate [1/h]	k^A^_ini_ k^B^_ini_	10–1000 (log)	5–500 (log)	5–500 (log)	5–500 (log)	Fixed from simulations Fig. 2
Pol II pause release rate [1/h]	k^A^_rel_ k^B^_rel_	10–1000 (log)	100,000	100,000	100,000	100,000
probability of proximal termination	p^A^_term_ p^B^_term_	0.05–0.9 (log)	0	0	0	0
Average stability of AS-induced OFF state A [h]	t^A^_OFF−AS_	1/3600–10 (log)	1/3600–1/60 (log)	1/3600–1/60 (log)	1–10 (log)	Fixed from simulations Fig. 2
Average stability of AS-induced OFF state B [h]	t^B^_OFF−AS_	1/3600–10 (log)	1/3600–1/60 (log)	1–10 (log)	1–10 (log)	Fixed from simulations Fig. 2
Number of OFF states	n	1–5 (lin)	1	1	1	1
Overlap length [kb]	L	0.5–50 (lin)	0.5–50 (lin)	0.5–50 (lin)	0.5–50 (lin)	Fixed from simulations Fig. 2
PR probability	p_PR_	1	0–1 (lin)	0–1 (lin)	0–1 (lin)	Fixed from simulations Fig. 2
collision probability	p_coll_	1	0–1 (lin)	0–1 (lin)	0–1 (lin)	Fixed from simulations Fig. 2
Average basal OFF time [h]	t^A^_OFF−basal_ t^B^_OFF−basal_	-	-	-	-	0.01–100 (log)
Average basal ON time [h]	t^A^_ON−basal_ t^B^_ON−basal_	-	-	-	-	0.01–100 (log)
Elongation rate [bp/s]	k_elong_	40	40	40	40	40

The model assumes two mechanisms of repression between the convergent genes: (1) Transcription-dependent promoter repression through modification of the promoter-associated chromatin state and (2) transcriptional interference, occurring when Pol II complexes on two convergent strands meet. We differentiate between (i) encounters of two elongating Pol II complexes (collisions) and (ii) encounters between a promoter-bound Pol II and an elongating Pol II (sitting-duck-interference). Collisions result in dislodgement of one randomly chosen Pol II, while sitting-duck-interference always results in the removal of the promoter-bound Pol II. All of these mechanisms have been experimentally observed, and modeled in several previous studies [6, 10, 16–18, 31–41].

To simulate a transient signal, which induces one of the genes (and thus indirectly represses the other), we start the simulation from two alternative initial expression states (Ini1, Ini2). In Ini1, Gene A is transcribed and Gene B is repressed, while in Ini2 a transient signal has induced Gene B leading to repression of Gene A (Fig. 1B). We performed the simulation in a stochastic manner using the Gillespie algorithm (only Pol II elongation was modeled deterministically) with more than 35,000 randomly chosen parameter sets. We combined a range of transcription strengths and locus features, to test whether – and under which conditions – the model could produce expression memory (Table 1, Full model). The parameter ranges tested were based on previous experimental estimates and should thus represent physiologically relevant regimes (see methods section for details). For each parameter set, the simulation was performed 100 times.

For a subset of parameter values, the model exhibited memory over several days (example simulation in Fig. 1D, top). Notably, the transitions between the two expression states typically occurred in a switch-like manner, suggesting a bistable system. To quantify the stability of each expression state, we extracted the first switching times (FST), defined as the time that elapses before the initially repressed gene becomes dominant (Gene B for Ini1 and Gene A for Ini2, Fig. 1B, D, see methods for details), and then averaged the FST across all simulated alleles. To identify parameter sets that can stably maintain both states, we extracted the minimal FST (minFST) for each locus, given by the average FST of the less stable state. We then classified parameter sets into those displaying no (minFST < 10 h), short-term (10 h < minFST < 100 h), and long-term (minFST > 100 h) memory. While > 90% of tested parameter sets exhibited no memory, 5.7% and 0.5% showed short-term or long-term memory, respectively (Fig. 1E). This demonstrates that stable memory (days-weeks) can arise from the combination of mechanisms that occur on timescales of seconds to hours (Table 1).

To understand which inhibitory mechanisms and kinetic reactions were required to stabilize both states, we tested 10 model simplifications, removing one mechanism at a time (Fig. 1F, Additional file 1: Table S1). Removing antisense transcription-induced promoter repression completely abolished expression memory. Also, omission of transcriptional interference by collisions substantially reduced the average memory timescale and thus the percentage of parameter sets generating long-term expression memory. These results suggest promoter repression, and to a lesser extent polymerase collisions, as the main mechanisms underlying expression memory at antisense loci, which could stabilize alternative expression states several weeks under certain conditions.

### Antisense pairs with promoter overlap can maintain alternative expression states for days to weeks

**Fig. 2:**
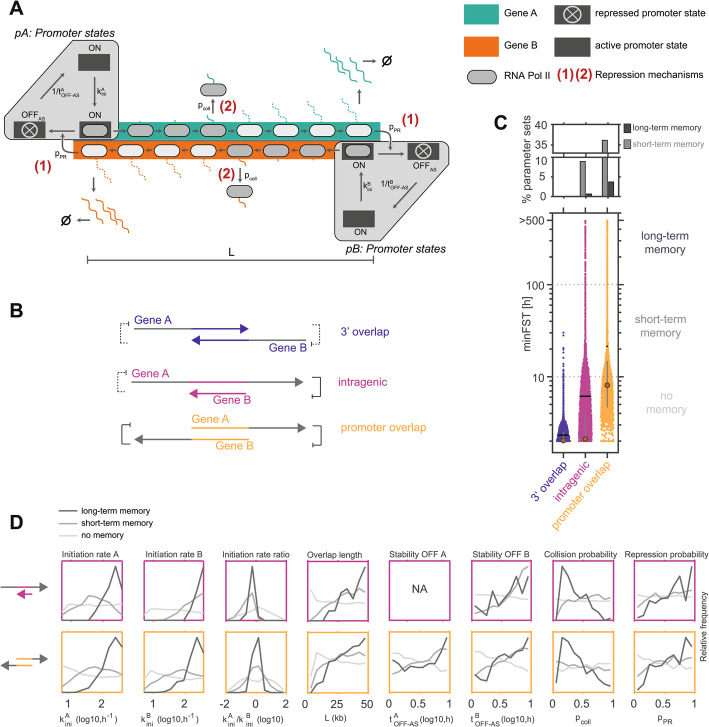
Long-term expression memory at antisense loci requires promoter repression. **A** Scheme of maximally simplified model version with promoter repression (1) and polymerase collisions (2), one-step initiation, and a single promoter OFF state. **B** Scheme depicting possible antisense transcription architectures including 3’ (blue), intragenic (pink) and promoter (yellow) overlaps. **C** Distribution of minFST for different locus architectures (bottom) together with percentage of parameter sets displaying short-term (gray) and long-term (dark gray) expression memory (top). **D** Distribution of parameter values for intragenic (top) and promoter overlap (bottom) architectures, across parameter sets displaying no (light gray), short-term (gray), and long-term (dark gray) expression memory

We next wanted to understand the precise requirements for expression memory. Here, we focused on the most simplified model, which retained promoter repression and polymerase collision, but contained only one promoter OFF state, did not account for pausing, premature termination and sitting-duck-interference (Fig. 1F, right, Fig. 2A). We resimulated the model with more parameter sets (119,000 sets in total). To account for variable efficiency in promoter repression and collisions we additionally varied the frequency with which promoter repression and collisions occur (Table 1). The strength of promoter repression was now controlled by two parameters: p_PR_ denotes the probability that a promoter switches to the OFF state, when transcribed by an antisense polymerase, and t_OFF−AS_ defines the stability of the repressed state as the time that elapses before the promoter turns back ON. To account for promoter-specific stability of the repressed state, we treat t_OFF−AS_ as a promoter-specific parameter. For most simulations we assume that p_PR_ is identical for both promoters, but this assumption does not affect the results (Additional file 2: Fig. S1).

We divided the simulated parameter sets into three categories that correspond to different locus architectures (Fig. 2B, Table 1). Pairs, where the gene bodies overlap, but transcription does not proceed through the opposite promoter, were termed ‘3’ overlap’. When transcription through the antisense promoter induces gene repression, the parameter set was categorized as ‘promoter overlap’, while pairs where one gene resides within another were termed ‘intragenic’. To compare the memory capacity between locus architectures, we again extracted the minFST for each parameter set. Pairs with promoter overlap had the highest memory potential, followed by intragenic overlaps, while 3’ overlapping pairs could not stabilize alternative expression states for extended time periods (Fig. 2C). These results are consistent with our finding that promoter repression is essential for long-term expression memory (Fig. 1F).

To better understand what allows certain parameter sets to stabilize alternative expression states, we analyzed the distribution of parameter values among the sets maintaining no, short-term, and long-term expression memory for the intragenic and promoter overlap architectures (Fig. 2D). The most stable sets exhibited frequent and stable promoter repression (high values for p_PR_ and t_OFF−AS_), again pointing towards the importance of this repression mode. With respect to the second repression mechanism mediated by polymerase collisions, memory was most stable for parameter sets with a long overlap (high L, allowing more collisions), and a surprisingly low collision probability (p_coll_ ~0.35). Although collisions must occur for stable memory, they should not be too frequent, potentially to still allow polymerases to reach the convergent promoter. Finally, stable memory was typically found for strong promoters (high k_ini_), probably required for efficient repression of the convergent gene through collisions and promoter repression. While for pairs with mutual promoter overlap, the initiation rates of the two promoters had to be tightly balanced for stable memory, at intragenic pairs the embedded gene (Gene B) required a stronger promoter, potentially because it had to rely solely on collisions to repress the convergent gene (Fig. 2D, top). Overall, the ability to repress the convergent transcript thus seems to be important for each strand to stabilize its transcribed state.

Taken together, our model analysis shows that mutual repression of antisense gene pairs could stably maintain transcriptional states for days to weeks, in particular if transcription-induced promoter repression is sufficiently strong. Although the maximal stability of the repressed promoter state we assumed was only 10 h, this could give rise to memory within the timescale of weeks, because antisense transcription continuously reinforces promoter repression. This represents a temporal amplification, where short-term promoter memory is extended into longer-term gene expression memory through an antisense locus architecture.

### Transcriptional bursting and mitosis destabilize antisense memory

**Fig. 3:**
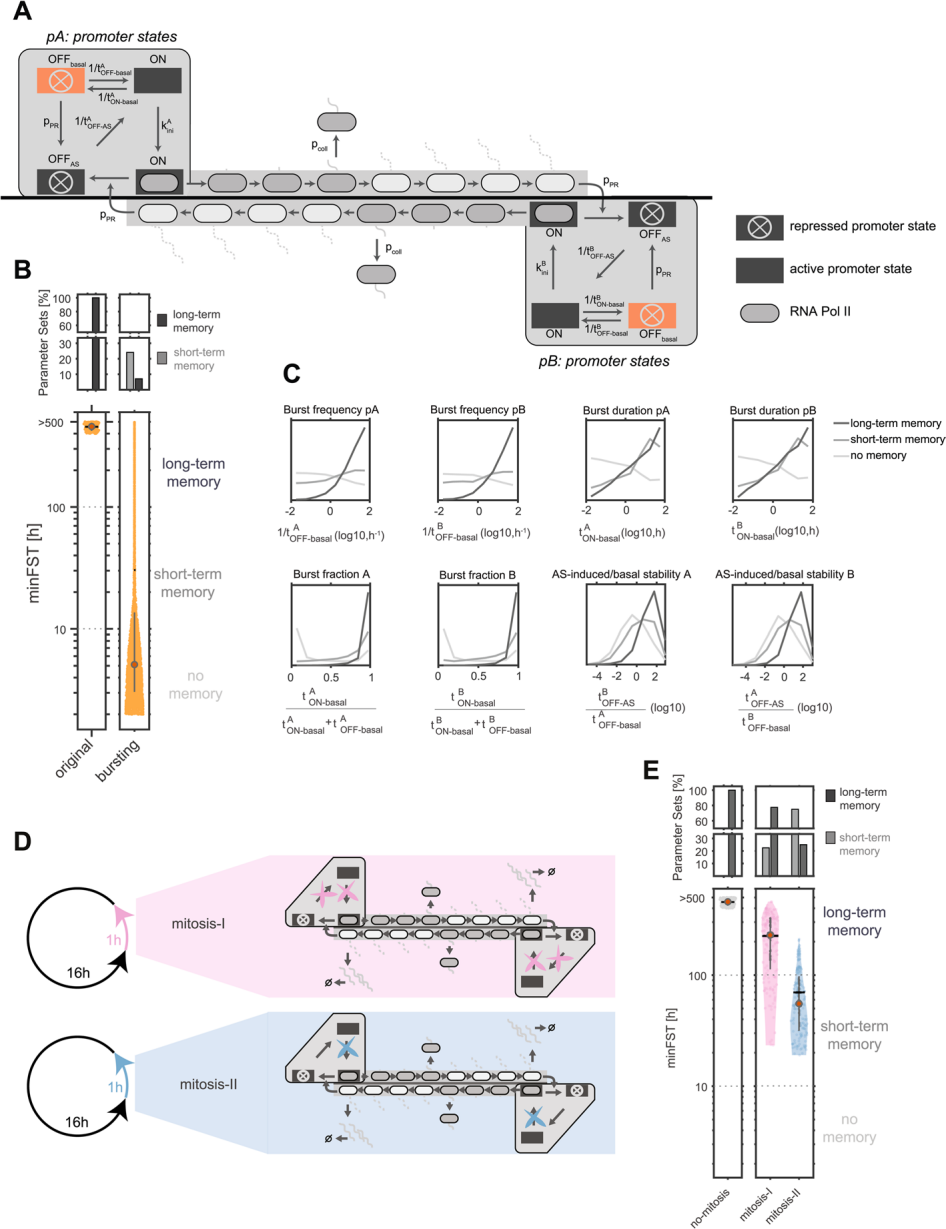
Transcriptional bursting and mitosis constrain but do not eliminate long-term expression memory. **A** Scheme of model version accounting for transcriptional bursts where the promoter can spontaneously transition to a basal OFF state that is distinct from the antisense transcription-induced OFF state. **B** Distribution of minFST for original and bursting model (bottom) together with percentage of parameter sets displaying short-term (gray) and long-term (dark gray) expression memory (top). **C** Distribution of transcriptional burst parameters, across parameter sets displaying no (light gray), short-term (gray), and long-term (dark gray) expression memory. **D**, **E**) Expression memory at antisense loci in the presence of mitosis. **D** Scheme of two alternative implementations of mitosis. Mitosis occurs every 17 h, lasting 1 h, during which transcription initiation is blocked. In scenario 1 (pink), antisense-induced PR is irreversible during mitosis; in scenario 2 (blue), PR can be reversed. **E** 160 parameter sets displaying long-term memory (> 400 h) without mitosis were simulated with the two mitosis scenarios. Distribution of minFST (bottom) together with percentage of parameter sets displaying short-term (gray) and long-term (dark gray) expression memory (top)

In our models transcription stabilizes memory by reinforcing repression of the convergent promoter. Transcriptional bursting, which results in discontinuous transcription, or mitosis, where transcription is interrupted, are thus likely to destabilize memory. We therefore extended our simulations to evaluate how these processes influence gene expression memory (Fig. 3).

Mammalian transcription is known to occur in stochastic bursts, with periods of active transcription interspersed with transcriptionally inactive periods [29, 42]. To account for transcriptional bursting we extended the model by including a basal OFF state (Fig. 3A, see methods for details). Bursting dynamics are described by two additional parameters, namely the stability of the basal OFF state (t_OFF−basal_) and the stability of the ON state in the absence of antisense-induced repression (t_ON−basal_). We resimulated the parameter sets that were most stable in the original model without bursting (memory >400 h), combining each of them with 200 randomly sampled values for the bursting parameters (Fig. 3A; Table 1). Compared to constitutive transcription, the presence of bursting substantially reduced the memory capacity of a locus (Fig. 3B). However, long-term memory still emerged for a subset of parameters (7%), in particular when bursts occurred with sufficiently high frequency (1/t_OFF−basal_) and duration (t_ON−basal_), or when antisense transcription-induced repression of the convergent promoter (t_OFF−AS_) was stable enough to persist through basal OFF periods (e.g. t^B^_OFF−AS_ > t^A^_OFF−basal,_ Fig. 3C). In summary, our analysis shows that antisense-mediated memory can persist for bursting gene pairs in a specific bursting regime, with memory stability being strongly dependent on the bursting time scales.

We next investigated how memory stability is altered by mitosis, which is not included in our original model and where transcription initiation is paused [43, 44]. We again resimulated the most stable parameter sets (memory >400 h), this time assuming that mitosis occurs deterministically every 17 h and lasts for 1 h, during which no new polymerases can initiate transcription (k_ini_=0). We considered two scenarios: one in which repression was maintained during mitosis, and another in which it was reversed at rates similar to interphase (Fig. 3D). In both cases, memory was destabilized but to different extents (Fig. 3E). Without reversal (mitosis-I), memory remained in the range of weeks (median 230 h), while repression reversal (mitosis-II) reduced memory duration more strongly (median 55 h). Previous work has shown that repressive chromatin modifications appear to remain stable throughout mitosis [45], specifically supporting the first, more stable, scenario. Moreover, recent studies suggest that TF binding, DNA accessibility, and even low-level transcription can persist to some extent during mitosis [46–48]. These findings suggest that cells may partially retain their transcriptional state through mitosis, which would further mitigate its destabilizing effect on memory. Mitosis is thus expected to reduce memory duration, but would still allow long-term expression memory in the range of weeks.

These results collectively indicate that despite the challenges posed by mitosis to gene expression memory maintenance, long-term memory can indeed be sustained in cycling cells. In addition, we would expect memory capacity to be significantly extended in post-mitotic cells.

### Genome-wide analysis detects skewed transcription at promoter-spanning antisense loci

Having shown that antisense gene pairs can in theory exhibit gene expression memory, we next set out to test whether mutual repression at antisense loci indeed occurs *in vivo.* To characterize endogenous antisense transcription in a genome-wide fashion, we re-analyzed a time-course experiment we had previously performed in female mESCs at days 0, 2 and 4 of differentiation by 2i/LIF withdrawal [49]. We reasoned that a joint analysis of different time points of this major cell state transition should allow us to observe dynamic changes in the expression states of antisense loci.

**Fig. 4:**
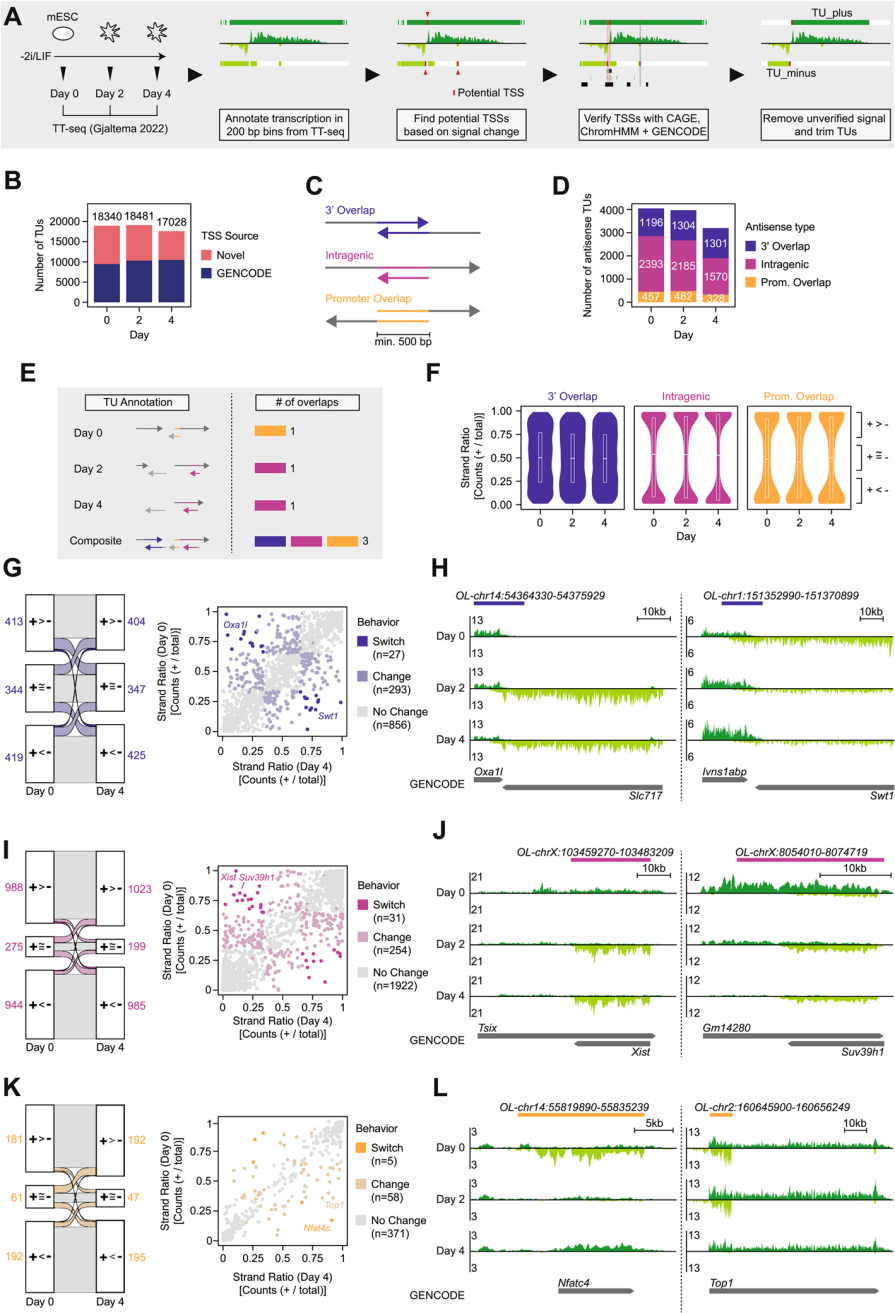
Genome-wide analysis of nascent transcription efficiently detects widespread antisense transcription. **A** Scheme of nascent transcriptome assembly. TT-seq data from day 0, 2 and 4 of differentiation was used to annotate transcription genome-wide. Afterwards, potential TSSs were detected based on strong increases in transcription and verified by chromHMM, GENCODE and CAGE data [53–56]. **B** Number of annotated transcribed regions per time point. Transcribed regions whose TSS overlaps with a GENCODE transcript are shown in blue, others are shown in red. **C** Scheme depicting possible architectures of antisense transcription including 3’ overlaps (purple), intragenic overlaps (pink) and promoter overlaps (yellow). **D** Number of overlaps that were found using the assemblies of days 0, 2 or 4 of differentiation. The total number of overlaps per type is indicated on the respective bar in white. **E** Scheme depicting the generation of a composite overlap set. The assemblies of 0, 2 and 4 days of differentiation were merged together and common overlaps called between all conditions. Overlaps shorter than 500 bp were discarded and intersecting overlaps combined. **F** Strand ratio within the composite overlap set separated by day and overlap type. The ratio was calculated as the fraction of total counts mapping to the plus strand. **G** Sankey diagram and scatter plot depicting the strand ratio of 3’ overlaps at day 0 versus day 4. For each time point, an overlap was defined as biased, if transcription was significantly different between the two strands across *n* = 2 biological replicates (Student’s T-test, Benjamini-Hochberg correction, FDR < = 0.1) and the strand ratio was >= 0.65 (plus bias) or < = 0.35 (minus bias). An overlap was annotated as ‘switch’, if it changed from one bias to the other and as ‘change’ if it changed from no bias to bias (or vice-versa). In the Sankey diagram, the total number of overlaps in each condition are depicted next to the boxes. **H** Genome browser screenshot showing TT-seq data for two switching 3’ overlaps. Reads from the plus-strand are colored in dark green, while reads from the minus-strand are shown in light green. Overlap annotation is shown above the tracks. **I** As in (**G**), but for intragenic overlaps. **J** As in (**H**), but for intragenic overlaps. **K** As in (**G**), but for promoter overlaps. **L** As in (**H**), but for promoter overlaps. Notably, the discovered antisense transcripts are not part of the ENCODE annotation

To detect antisense transcription we used Transient-Transcriptome sequencing (TT-seq) data, which profiles nascent transcription [50]. TT-seq is based on short pulse-labeling of newly produced RNA combined with transcript fragmentation and therefore detects the entire transcribed region. Consequently, the method allows sensitive detection of lowly expressed and unstable transcripts, which are often involved in antisense transcription [51]. To study antisense transcription beyond annotated genes, we first assembled a nascent transcriptome annotation for each time point from scratch (Fig. 4A, see methods for details), as done previously for such data [52]. We segmented the genome into transcribed and non-transcribed 200 bp fragments for each strand. We detected putative TSSs as abrupt changes in nascent transcription (>10x fold change). As *bona fide* TSSs, we then defined those that overlapped with a previously annotated TSS (GENCODE or mESC CAGE data) or with a promoter/enhancer state detected by chromatin segmentation (ChromHMM) based on measurements of DNA accessibility and active histone marks in the same cellular context [49, 53–56]. Lastly, we removed any sporadic transcription without a verified TSS, trimmed the resulting transcription units (TUs) to a 10 bp resolution and merged those that overlapped on the same strand. In doing so, we recovered 17,028 − 18,481 TUs per time point, approximately half of which were not part of the commonly used GENCODE annotation and were intergenic, likely non-coding, transcripts (Fig. 4B, Additional file 2: Fig. S2A-E, Additional file 3: Table S2).

To identify antisense loci, TUs on different strands that overlapped by at least 500 bp were detected and grouped into promoter, 3’ and intragenic overlaps (Fig. 4C-D). Intragenic and promoter overlaps were shorter on average (3542/2716 bp) compared to 3’ overlaps (8978 bp, Additional file 2: Fig. S2F). While 3’ overlaps frequently occurred between coding genes, potentially representing read-through transcription, promoter and intragenic overlap pairs typically consisted of a coding and a non-coding TU (Additional file 2: Fig. S2G). TUs with promoter overlaps, but not those with 3’ overlaps, were enriched for specific gene ontology terms (Additional file 2: Fig. S3A). Many of the identified terms are associated with regulatory processes, such as chromatin organization and RNA splicing.

At each time point, 5,734-6,991 TUs were involved in at least one antisense pair. In mESCs and after 2 days of differentiation, we detected ~ 4,000 antisense pairs, which was reduced to ~ 3,200 at day 4. Interestingly, only the number of detected intragenic and promoter overlaps was reduced, while the number of 3’ overlaps remained stable (Fig. 4D). This means that loci where antisense transcription over a TSS is detected together with transcription from that TSS become less frequent upon mESC differentiation. One interpretation could be that antisense-mediated repression becomes more potent when mESCs differentiate.

Since our analysis so far treated each time point separately, we could not detect transcript pairs that are expressed in a mutually exclusive manner, where the dominant TU might switch upon differentiation. We therefore assembled a set of composite overlaps by combining the assemblies across the time course (Fig. 4E). After removing lowly expressed gene pairs, a set of 3817 antisense loci was identified, containing 434 promoter, 2207 intragenic and 1176 3’ overlaps. To investigate whether antisense transcripts show signs of mutual repression, we quantified to what extent they were expressed in a mutually exclusive manner. To this end, we defined a strand ratio as the fraction of TT-seq reads in the overlapping region that map to the plus strand (Fig. 4F). While transcription at intragenic and promoter overlap transcript pairs was strongly biased towards one of the strands (strand ratio close to 0 or 1), which became even more pronounced during differentiation, TUs in 3’ overlaps were often expressed at similar levels (strand ratio ~ 0.5). A similar trend was observed when analyzing the time-point specific transcript annotation instead of the composite overlaps (Additional file 2: Fig. S3B). Notably, mutually exclusive expression was more common in overlaps with high total transcription (Additional file 2: Fig. S3C). This finding suggests that transcription through a promoter indeed mediates gene repression, which seems to lead to switch-like behavior at antisense loci.

To test whether antisense-mediated repression might have a functional role during mESC differentiation, we next identified loci, where the dominant strand switched during the time course (Fig. 4G-L, Additional file 2: Fig. S3D-F, Additional file 3: Table S2). While the strand ratio remained unchanged for most antisense pairs, we detected a small set of loci (5–31 antisense pairs) for each locus architecture that switched the dominant strand (strand ratio >0.35 to < 0.65 or vice versa). These include known antisense pairs such as *Xist/Tsix* and *Suv39h1/Suv39h1as* [57, 58], as well as loci that to our knowledge have not been implicated in antisense-mediated regulation in the past, for example *Nfatc4* and *Top1* (Fig. 4G-L). For these switching loci, antisense-mediated repression might be involved in their regulation during early mESC differentiation. Antisense pairs that are stable in our analysis might nevertheless switch in other cellular transitions.

### Promoter repression via antisense transcription correlates with increased DNA methylation

**Fig. 5:**
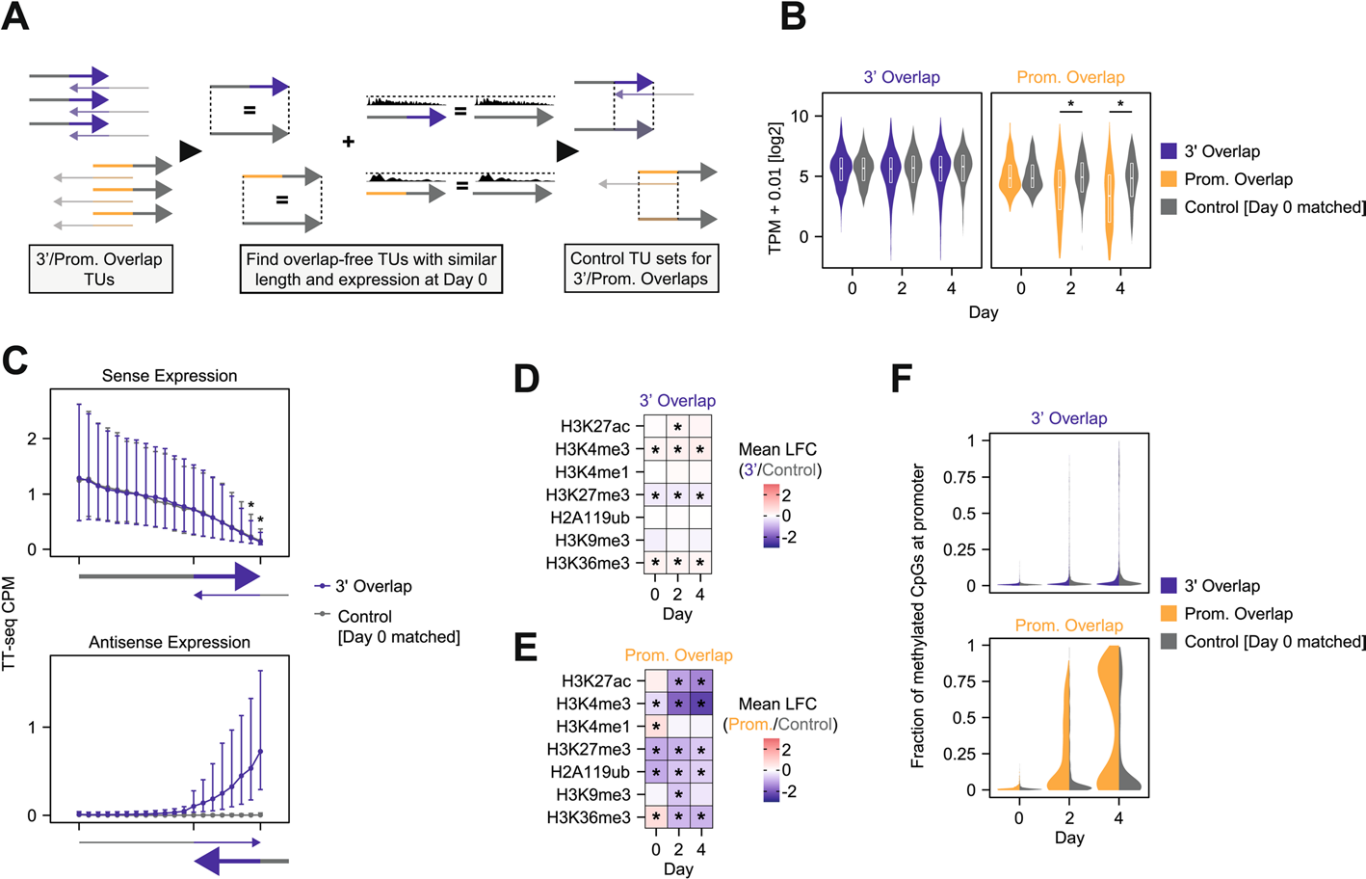
Promoter-overlapping antisense transcription primes for repression during differentiation. **A** Scheme depicting the generation of a set of overlap-free control genes. **B** Expression measured by TT-seq at 3’ and promoter-overlapping transcribed regions annotated based on transcription at day 0 in comparison to matched controls. Significance was assessed using a ranked Wilcoxon sum test (p < = 0.01). **C** Binned line plot showing sense or antisense expression of TT-seq data in 3’ overlap transcribed regions and mock-overlapping controls at day 0. Expression was quantified in 12 “free” bins and 8 “overlapping” bins. The big dots depict the median of all transcribed regions, while the upper and lower whiskers depict 3rd and 1st quartiles respectively. Significance was assessed using a ranked Wilcoxon sum test (*p* < = 0.01). D-E) Heatmap depicting mean log2 fold change of different histone marks at promoters between 3’ overlapping transcribed regions (**D**) or promoter overlaps (**E**) compared to overlap-free controls. The CUT&Tag data was taken from [49]. * Significance was assessed using a ranked Wilcoxon sum test (*p* < = 0.01). **F** Violin plot depicting the percentage of promoter CpG methylation in 3’ or promoter overlapping TUs. Methylation levels for corresponding overlap-free controls (matched at day 0) are shown as gray diamonds. Promoters were defined as 500 bp upstream to 200 bp downstream of a TSS

As our initial analyses identified mutually exclusive antisense transcription primarily at loci overlapping promoter regions, we examined the molecular mechanisms governing antisense transcription-mediated repression in more detail. We focused on 3’ overlaps to investigate polymerase collisions, and promoter overlaps to analyze antisense transcription-induced promoter repression. In the first step, we defined a set of overlap-free control TUs with matched length and nascent RNA expression for each antisense pair detected at day 0 for each locus architecture (Fig. 5A, Additional file 2: Fig. S4A, Additional file 4: Table S3) and compared their transcriptional activity throughout the time course to antisense TUs (Fig. 5B). TUs with 3’ overlap remained expressed at similar levels as the controls throughout the time course. TUs in promoter overlaps by contrast were more frequently downregulated upon differentiation compared to overlap-free controls (median fold change 0.30 compared to 0.95 in controls). When performing the same analysis for antisense pairs detected at day 4 with controls matched on day 4 expression, again controls exhibited little change across time points, while expression at antisense pairs increased over time (Additional file 2: Fig. S4B-C). The extent of upregulation, however, was smaller compared to the Day 0-centered analysis (median fold change 0.49 compared to 0.92). This observation suggests that antisense loci exhibit more dynamic expression than overlap-free genes. Moreover, the strong downregulation of antisense pairs expressed in undifferentiated cells might suggest that convergent transcription through a promoter becomes more repressive upon mESC differentiation. Antisense transcription in the gene body by contrast (3’ overlap), which cannot be mediated by promoter repression and might instead involve polymerase collisions, exhibited no sign of repressive activity in this analysis.

To more directly probe for transcriptional interference by polymerase collisions, we tested whether the TT-seq signal would decay in the overlapping part of 3’ overlap pairs more rapidly than control genes. We analyzed transcription activity along the transcripts in the overlapping and not overlapping region and compared them to overlap-free control TUs (for details see methods section). The distribution of transcription along the gene was very similar to control genes, albeit a slight reduction in the signal was observed towards the end of the transcript (Fig. 5C, Additional file 2: Fig. S4D). This suggests that transcriptional interference by polymerase collision is weak, or only present in a small subset of genes.

Having observed indications of antisense transcription-mediated promoter repression, in particular during differentiation, we asked which mechanisms could mediate the repression. We therefore examined the chromatin state at the promoters of antisense TUs. We re-analyzed a CUT&Tag dataset of histone marks that we had generated previously at the same time points as the TT-seq data [49] and performed whole-genome bisulfite sequencing (WGBS) under the same conditions to analyze DNA methylation (see Additional file 2: Fig. S4E for example tracks).

As expected, we found only very small differences at promoters of 3’ overlapping TUs (where transcription does not extend through the promoter) compared to the overlap-free controls (Fig. 5D). For promoter-overlapping transcripts by contrast we found several differences (Fig. 5E-F). At day 0, promoters exhibited higher H3K36me3 levels, a mark deposited co-transcriptionally, which has previously been implicated in antisense-mediated repression [59]. At all time points, H3K4me3 levels were decreased in antisense promoters relative to controls. Among the repressive marks, only DNA methylation was enriched at antisense-transcribed promoters upon differentiation (Fig. 5F). H3K9me3 on the other hand, which is thought to become enriched at the *Xist* locus in response to antisense transcription [49, 60], was not globally associated with promoters repressed by antisense transcription, rather the opposite. The levels of H3K27me3 and H2AK119ub were even recorded below the overlap-free control group. As these marks are reportedly removed by transcription [61, 62], this finding is not entirely surprising.

Taken together, our genome-wide analysis shows that antisense loci, where at least one partner transcribes through the promoter of the other one, exhibit signs of mutual repression and switch-like behavior. These loci typically consist of a protein-coding gene associated with regulatory processes and a non-coding antisense gene, pointing towards a regulatory role of antisense transcription at these loci. These findings are in line with our model analysis that proposed promoter repression to be the main determinant of memory, which is associated with switch-like behavior. With respect to polymerase collisions we find little experimental support in our genome-wide analysis, which could suggest that they occur rarely or only in a subset of loci. Finally, we find evidence for an increase in antisense-mediated repression strength during differentiation, potentially due to deposition of DNA methylation. As a consequence, mutual repression at antisense pairs becomes more pronounced during differentiation and we detect fewer pairs where both strands are transcribed simultaneously.

### Antisense transcription induces stable promoter repression during differentiation

**Fig. 6:**
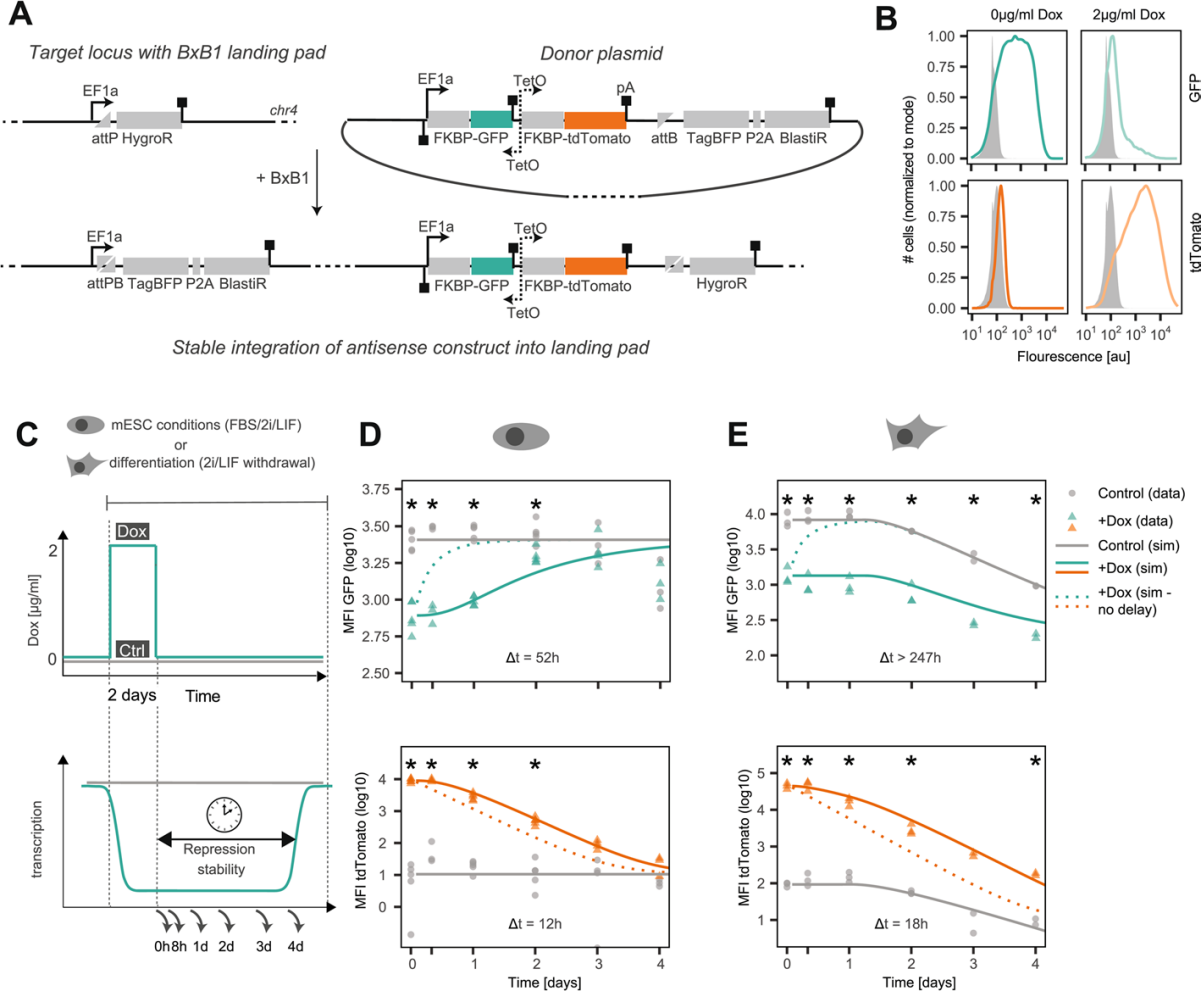
Antisense transcription can induce stable repression in a synthetic antisense construct upon differentiation. **A** Stable integration of synthetic antisense construct into genomic locus carrying a Bxb1-landing pad. Structure of the antisense construct developed in this study: human EF1a promoter drives transcription of a FKBP-coupled GFP. A bidirectional doxycycline-inducible TetO promoter drives convergent transcription across the GFP TU, and simultaneously transcribes a FKBP-coupled tdTomato reporter. Arrows and square boxes indicate promoters and polyadenylation signals, respectively. **B** Flow cytometry profiles of the TxSynAS line cultured for 2 days in the presence or absence of doxycycline. **C** Scheme of doxycycline treatment profiles used in **D** and **E**. For two days, cells were either kept in medium without (control), or with high Dox concentrations (+ dox). Cells were then again cultured in the absence of doxycycline and reporter levels were assessed at the indicated time points by flow cytometry. **D**,** E** Background-corrected mean fluorescence intensity (MFI) of GFP (top) and tdTomato (bottom) in mESC medium FBS/2i/LIF (**D**) and upon differentiation by 2i/LIF withdrawal (**E**) for the experimental setup shown in C (circles, triangles). Difference in expression levels between the two initial conditions was assessed with an unpaired Student’s t-test (*p* < 0.05, asterisks). Lines represent simulation of the ODE model for the control data (gray), for + dox with the reactivation delay Δt that best explains the data (colored solid), and for immediate reactivation (dotted colored). Simulations were performed with the parameter values that best explain the data (see Additional file 5: Table S4)

Since our results point towards a central role of promoter repression, we decided to experimentally assess the efficiency and stability of antisense transcription-induced promoter repression through a synthetic biology approach. We designed a synthetic antisense locus that was stably integrated into the genome of female mESCs through a BxB1-dependent landing pad system, and named the resulting cell line TxSynAS (Fig. 6A). The synthetic nature of the construct allows us to monitor expression of both strands via fluorescent reporters and investigate the emerging regulatory responses independent of a functional role of the encoded transcripts or proteins. In the sense direction, a constitutively active EF1a promoter drives transcription of a GFP TU. For transient induction of antisense transcription, we utilized a commonly used Doxycycline-inducible bidirectional promoter (pTRE-Tight, Fig. 6A), which drives antisense transcription through the GFP TU in one direction and transcribes a fluorescent reporter (tdTomato) to monitor antisense promoter activity in the other direction. This setup allows modulation of antisense transcription and assessment of its repressive effect on sense expression. Unlike a full toggle switch, our system includes only one repression arm: while antisense transcription represses sense expression, the antisense promoter is externally controlled and unaffected by sense transcription.

In the absence of Dox, cells express GFP, but not tdTomato (Fig. 6B, left). Treatment with Dox led to upregulation of tdTomato and a reduction in GFP expression (Fig. 6B, Additional file 2: Fig. S5A). These observations confirm that transcription from the convergent promoter represses expression of the sense strand, and that our assay can detect such antisense transcription-mediated repression.

To quantify the stability of transcription-induced repression, we treated cells for 2 days with Dox (2 µg/ml) or without (control) and followed sense and antisense expression by flow cytometry over time for another 4 days (Fig. 6C). Since our genome-wide analysis had indicated that antisense-mediated promoter repression might be more potent upon differentiation, we performed the experiment in undifferentiated and differentiating conditions (2i/LIF withdrawal). In ESC conditions, GFP and tdTomato levels converged towards the same levels as the control at the end of the experiment (Fig. 6D). During differentiation by contrast, repression appeared to be sustained for a longer time (Fig. 6E). However, even in the absence of Dox, GFP expression gradually decreased during differentiation, complicating interpretation of the results. This might be due to downregulation of the EF1a promoter or silencing of the landing pad during differentiation, as the effect is conserved across different genomic integration sites (Additional file 2: Fig. S5B-E).

To quantify the stability of antisense-induced repression we used mathematical modelling. We developed a simple ordinary differential equation (ODE) model describing the GFP promoter states (ON, OFF) and production and degradation dynamics of GFP protein (Additional file 2: Fig. S5F, see methods for details). We first estimated the rates of protein dilution through cell division and protein degradation for our constructs, based on independent measurements and estimates from the literature (see methods for detail). Using the control time course we then estimated the parameters associated with differentiation-dependent GFP production dynamics (Fig. 6E, grey line, Additional file 2: Fig. S5G). To assess whether repression of the sense gene was maintained after Dox removal, we first simulated reporter expression upon Dox withdrawal, assuming immediate GFP reactivation (Fig. 6D-E, dotted lines). Reactivation occurred more slowly in our experimental data compared to the model simulation under both conditions (Fig. 6D-E), showing that GFP promoter repression is maintained after Dox washout. Notably, this effect was much more striking during differentiation.

To quantify the stability of antisense-induced GFP repression, we allowed for a delay before reactivation by assuming that the promoter transitions through several intermediate states before reaching the final ON state. By fitting the delay-associated parameters to the experimental data we estimated repression stability. As a control, we performed an analogous analysis for the tdTomato inactivation, to estimate effects of incomplete Dox washout. In undifferentiated cells, a delay of 52 h had to be assumed for GFP, while the delay for tdTomato was substantially shorter (12 h) (Additional file 5: Table S4). The fact that the delay observed for GFP is 30 h longer than the one observed for tdTomato, suggests that antisense-mediated promoter repression could persist for several hours up to a day in ESCs. During differentiation, tdTomato repression was similarly fast (17 h), but a large delay of > 247 h had to be assumed to reproduce GFP dynamics. This shows that antisense transcription-induced repression of the sense reporter becomes stabilized upon differentiation and that the repressed state can persist over several days.

In summary, the experimental results support our theoretical model and genome-wide analysis, by demonstrating that antisense transcription can induce stable promoter repression, in particular during differentiation. Our data demonstrates that repression can be stable for hours to days, while our modelling analysis predicted stable memory for promoter repression in the range of 2–10 h. The experimentally observed timescales of promoter repression could thus enable antisense-mediated memory in the range of days to weeks, depending on cell type.

## Discussion

By combining three complementary approaches, we have dissected the conditions under which antisense transcription can give rise to expression memory. Through mathematical modeling and synthetic biology we showed that memory can be stable for several days, if RNA polymerases induce a long-lasting transcriptionally inactive chromatin state at the antisense gene promoter. This concept aligns with the observation of strong mutually exclusive activity at promoter-spanning convergent gene pairs from our genome-wide analysis, supporting the notion of transcription through the antisense promoter as repressive. Moreover, our findings suggest that the level of repression, and thus the potential for memory formation, is influenced by the cellular state. Repression appeared to be less pronounced in undifferentiated mESCs and strengthened as differentiation progressed, as evidenced by the detection of fewer co-transcribed antisense gene pairs with promoter overlap and stabilization of memory at the synthetic antisense locus.

Based on mathematical modeling we have established a quantitative framework to investigate the role of convergent transcription in generating *cis*-encoded expression memory. By systematically exploring various locus architectures and kinetic parameters, we discovered that enduring memory (lasting over 4 days) can be established when both genes exhibit high transcription rates (over 30 initiation events per hour) and trigger a repressed chromatin state with a lifetime in the range of hours. A recent genome-wide estimate of initiation rates in a mammalian cell line revealed a median rate of 30 h^− 1^ and 9 h^− 1^ for mRNAs and long non-coding RNAs, respectively [63]. In general, chromatin states can be stable for hours or even days [64] and the antisense-transcription associated H3K36me3 mark has a lifespan for ~ 1 h in yeast [65]. Our analysis thus predicts antisense-mediated *cis*-memory to occur in a physiologically relevant regime. It shows that a biological phenomenon (transcriptional memory) with kinetics in the order of weeks can arise from the combined action of multiple processes with timescales in the order of minutes to hours.

A key model parameter that determines memory stability is the lifetime of the antisense-induced repressed promoter state. Through construction of a synthetic antisense locus we showed that promoter repression can indeed arise from antisense transcription. The synthetic nature of our construct makes a regulatory role of the encoded transcripts highly unlikely, favoring a model in which the act of transcription mediates the establishment and maintenance of repression. While repression was only short-lived in mESCs, it became stabilized upon differentiation, in agreement with a previous study using a similar approach [34]. The fact that antisense-mediated repression was potent in all conditions, but stable maintenance of repression was restricted to differentiating cells, suggests that only a subset of repression mechanisms might be sufficiently stable to generate *cis*-memory. Work in yeast has established that antisense transcription induces histone deacetylation and loss of accessibility mediated by deposition of H3K36me3 [10, 31, 33]. Also, a study in mammalian cells, where Tsix, the antisense transcript of Xist was ectopically expressed in a cellular context where it is normally silent, revealed loss of active marks and accessibility as the earliest consequences of antisense transcription [60]. These changes were, however, rapidly reversible, and only the more slow acquisition of repressive chromatin marks, such as H3K9me3 and DNA methylation, established a memory at Xist when Tsix transcription was turned off.

The fact that our synthetic locus maintained only a short-term memory in mESCs suggests that repression is short-lived in that cellular context. Our genome-wide analysis revealed that promoters transcribed in the antisense direction acquire H3K36me3, but not DNA methylation or H3K9me3. The basic antisense-mediated repression mode present in yeast is thus already active in mESCs, but memory-forming mechanisms only become available upon differentiation. Similar observations were reported for other *cis*-memory systems. Transient inhibition of Polycomb repressive complex 2 (PRC2) was fully reversible in mESCs, while a subset of target genes retained a memory of transient PRC2 loss, when the same experiment was performed in a more differentiated cell type [66]. Similarly, transient epigenetic editing using the KRAB repressor domain, was found to be fully reversible in mESCs, but stabilized during differentiation [67]. The mESC-specific factor Dppa2 contributes to the reduced memory capacity in mESCs, but additional mechanisms likely exist [67]. An interesting correlation is that mESC differentiation is associated with a major increase in global DNA methylation levels [68, 69]. Whether an increased activity of *de novo* DNA methyl-transferases indeed underlies *cis*-memory at antisense loci remains to be investigated.

An interesting avenue for future work will be to elucidate the specific roles of different *cis*-memory systems and how they interact with *trans*-memory. An intriguing hypothesis is that they operate at different timescales. At the *Xist* locus, for example, antisense transcription is only present transiently for a few days before other, potentially more stable, mechanisms, such as DNA methylation, ensure permanent repression [70, 71]. Similarly, at the *FLC* locus in plants, antisense transcription can induce rapid silencing in response to external stimuli (temperature), which will later be consolidated by Polycomb repression [72]. The role of antisense-mediated repression as compared to purely chromatin-based systems might thus be to establish *cis*-memory for a shorter time frame, while remaining responsive to external stimuli. How the coupling of different mechanisms ensures responsive, but robust memory remains to be investigated.

## Conclusions

By integrating modeling, genomics, and synthetic biology, we uncovered how antisense transcription can generate cis-encoded expression memory. Our mechanistic model predicts that stable memory arises when convergent promoters drive high transcription and induce a repressed chromatin state with hour-scale persistence, which are physiologically possible conditions. Experiments confirm that antisense transcription can establish such repression, which becomes increasingly stabilized during stem cell differentiation. These findings reveal how multi-timescale molecular processes can produce lasting gene expression states and show that cells modulate their memory capacity as they exit the pluripotent state.

## Methods

### Simulations

#### Full antisense model

To simulate antisense transcription, initiation and elongation from two convergent promoters together with RNA degradation were combined into a mathematical model. Transcription initiation was modeled as a two-step process where Pol II complexes bind to the two convergently oriented promoters in a stochastic manner, and then are either released into productive elongation or spontaneously terminate transcription in the promoter-proximal region. Elongating Pol II complexes were moved along the respective gene in a deterministic manner in steps of 100 nt length. All other reactions were simulated using the stochastic Gillespie algorithm. For the elongation and RNA degradation rates, experimental estimates were used, as explained in the next section. Transcription of a Pol II complex through the convergent promoter segment induced a switch to a transcriptionally inactive OFF state. To account for experimental data that suggest a peaked waiting time distribution in the silent state for mammalian promoters [27–29], we modeled promoter reactivation as a multistep process, consisting of one active ON state where transcription can occur and n sequential inactive OFF states [25, 30]. When two convergent Pol II complexes occupied the same DNA segment, one randomly chosen Pol II was removed from the gene. If this segment was a promoter region, the promoter-bound Pol II was always removed from the DNA (sitting-duck-interference). Simulations were conducted in MATLAB_R2019b. The model was written in C + + and compiled into a MEX file that was called from the main MATLAB function. For parameter scanning, a compiled MATLAB script was executed in parallel on a computing cluster.

#### Simulating memory/maintenance of transcription state

To find parameter values that could maintain alternative transcription states at the simulated antisense locus, we first scanned a large parameter space. Degradation and elongation rates were set to fixed values based on previous experimental estimates of average rates in mammalian cells [22, 25, 26]. All other parameters were randomly sampled within realistic parameter ranges (Additional file 1: Table S1, Sheet par_ranges_full). We simulated >35,000 randomly sampled parameter sets.

To probe for conditions in which alternative transcription states at the antisense locus were stable, each allele was simulated twice starting from asymmetric initial conditions:

##### Initial condition 1

Gene A transcribed at steady state in the absence of antisense transcription-mediated repression, gene B not transcribed and promoter B OFF.

##### Initial condition 2

Gene B transcribed at steady state in the absence of antisense transcription-mediated repression, gene A not transcribed and promoter A OFF.

The steady state Pol II occupancy in the absence of transcriptional interference is calculated as:

$$\sum PolII = \frac{L_{Gene}}{k_{elong}}\cdot k_{net-ini}$$

Where $\frac{L_{Gene}}{k_{elong}}$ corresponds to the time that an elongating Pol II needs to transcribe the gene and $k_{net-ini} = \left(\frac{1}{k_{ini}} + \frac{1}k_{rel}\right) ^{-1} \cdot Pterm$ gives the net initiation rate, with $\frac{1}{k_{ini}} + \frac{1}{k_{rel}}$ corresponding to the average time the promoter needs to produce one elongating Pol II. The steady state RNA level can then be inferred as the ratio of net production and degradation rates:

$$\sum RNA = \frac{k_{net-ini}}{k_{deg-RNA}}$$

For each parameter set the antisense locus was simulated from both initial conditions for 500 h in 100 realizations.

As a classifier for the stability of the initial transcription state the first-switching-time (FST) was extracted. The FST denotes the first time point at which the initially inactive gene dominated transcription within the overlap. It was defined as the first time point at which the ratio of the sum of Pol II complexes within the overlap, normalized to the relative promoter strength of the two genes, crossed 1:

$$\frac{\frac{\sum PolII^{A}}{k_{net-ini}^A}}{\frac{\sum PolII^{B}}{k_{net-ini}^B}}$$

The FST was then averaged over all simulated alleles, and the minimum over the two initial conditions was used as a measure for transcriptional stability (minimal first-switching-time, minFST). A set was classified as exhibiting long-term memory if minFST > 100 h, and as exhibiting short-term memory if 100 h > minFST > 10 h.

#### Model simplifications

To analyze which interference mechanisms and transcription reactions were strictly required for the generation of expression memory, we generated five reduced model versions, each either lacking one of the transcriptional interference mechanisms or simplifying transcription initiation or promoter reactivation into single-step reactions.

- In the model without promoter repression, passing Pol II complexes do not affect the state of the opposing promoter.
- In the model without collisions, sense and antisense Pol II complexes can bypass one another.
- In the model without SDI, both Pol II complexes have the same probability to dislodge upon collision at the promoter segment.
- To simplify promoter reactivation into a single-step reaction, we set the number of promoter OFF states n to 1.
- To simplify transcription initiation into a single-step reaction, we set k_rel_ = 10^5^ h^− 1^ and p_term_ = 0 such that every Pol II that binds the promoter is immediately released into productive elongation. The new lumped one-step initiation rate k_ini_ was sampled between [5,500] h^− 1^ to preserve the average duration of transcription initiation.

Each simplified model was simulated with > 35,000 randomly sampled parameter sets. The simplifications that did not substantially reduce the fraction of parameter sets displaying long-term transcriptional memory, were then hierarchically combined to obtain a maximally simplified model structure (see Fig. 2A; Table 1 and Additional file 1: Table S1, Sheet FST_reduced_models).

We resimulated the maximally simplified model with a larger number of parameter sets (119,000). Since it is unclear how frequently Pol II complexes collide in vivo, or induce a repressed promoter state, we included two additional parameters p_PR_ and p_coll_ that modify the frequency of these events, respectively. Values for variable parameters were randomly drawn from uniform or logarithmic distributions (see Additional file 1: Table S1, Sheet par_ranges_simpl).

#### Simulating different antisense locus architectures

Simulations were classified into the three different locus architectures based on the stability of the repressed promoter state. An average OFF period of < 1 min was classified as unstable, while an average OFF period of > 1 h was classified as stable. Those parameter sets with unstable repression of both promoters were categorized as 3’ overlaps, since promoter repression for both genes is essentially absent. Those sets with unstable repression of one, but stable repression of the other promoter were categorized as intragenic architecture, where only the promoter of the embedded gene can be repressed. Finally, all sets in which both promoters could be stably repressed were assigned to the promoter overlap category.

#### Simulating transcriptional bursting

To account for bursty transcription, we introduced a basal promoter OFF state, distinct from the antisense-induced OFF state. The promoter spontaneously transitions between the ON and basal OFF states. When a Pol II transcribes through the convergent promoter region, it triggers a transition to the antisense-induced OFF state with probability pPR​, regardless of the promoter’s prior state. To evaluate the impact of bursting on memory stability, we combined all parameter sets stable for > 400 h in the non-bursty model with 200 randomly sampled burst parameter combinations.

#### Simulating mitosis

To assess the effect of mitosis on memory stability we assumed that mitosis occurs deterministically every 17 h and lasts for 1 h, during which no Pol II initiation occurs (kini = 0). Two scenarios were considered: one where antisense-induced promoter repression is stably maintained through mitosis, and another where it can revert at the same rate as in interphase. All parameter sets stable for > 400 h in the absence of mitosis were re-simulated under both scenarios.

#### Quantification of memory in the synthetic antisense construct

To quantify the timescale of antisense-induced promoter repression in the synthetic antisense construct, an ODE model was developed and model simulations were compared with the experimental data.

##### Quantification of reporter half lives

As a first step, degradation rates of FKBP-GFP and FKBP-tdTomato in the construct were estimated. The half life of wild type GFP has previously been determined to be 26 h [73], while tdTomato has been described to have a comparable or longer half life [74, 75]. To remain conservative in our modeling, we assumed the tdTomato half life to be equal to that of GFP, since underestimating tdTomato half life would lead to overestimation of the delay observed in the tdTomato dynamics. To estimate protein half lives of both reporters fused to the FKBP destabilization domain in the presence of 1µM stabilizing Shield1 ligand, we compared steady state expression levels of cell lines expressing the wildtype and FKBP-fused reporter version respectively, and found GFP to be destabilized 1.7x, and tdTomato 1.16x by fusion to the FKBP domain in presence of 1µM Shield1. With a half-life of 26 h for the wildtype proteins this results in a half-life of 15.3 h and 22.4 h for FKBP-GFP and FKBP-tdTomato, respectively.

##### ODE model of reporter kinetics for quantification of memory at the synthetic antisense locus

To quantify the stability of promoter repression after antisense transcription is terminated by Dox removal, an ODE model describing GFP dynamics was formulated. In the model, the GFP promoter can assume an inactive (OFF) or an active (ON) state. To reproduce potentially delayed reactivation of GFP, the transition from the OFF to the ON state is assumed to pass through n intermediate states (OFF_1…n_). GFP can only be produced from the ON state:

$${\frac{dOFF}{dt} = -k_{trans} \cdot OFF}$$

$${\frac{dOFF_{1}}{dt} = {k_{trans}} \cdot {(OFF-OFF_{1})}}$$

$$\frac{dOFF_{i}}{dt} = k_{trans} \cdot (OFF_{i-1} - OFF_{i}), {for}\ {i} = 2, 3, ..., n$$

$$\frac{dON}{dt} = k_{trans} \cdot OFF_{n}$$

$$\frac{dGFP}{dt} = k_{prod} \cdot {ON}-(k_{deg}+k_{dil})\cdot{GFP}$$

k_prod_ is the protein production rate, k_dil_ is the dilution rate during cell division and k_deg_ is the protein degradation rate. K_trans_ represents the transition rate between the promoter states.

The dilution rate is determined by the length of the cell cycle which in pluripotent mESCs has been measured to be roughly 12 h [76] as $k_{dil}=\frac{ln(2)}{12h}$, while the degradation rate is calculated from the protein’s half-life (estimated above) as $k_{deg-GFP}=\frac{ln(2)}{26} \cdot{1.7}$ and $k_{deg-tdT}=\frac{ln(2)}{26h} \cdot {1.16}$.The protein production rate was assumed to be constant in ESC conditions and set to $k_{prod}=k_{deg}+k_{dil}$, since the data was normalized between 0 and 1 (see below). During differentiation k_prod_ is assumed to change over time as follows:

$$k_{prod}=(k_{deg}+k_{dil}), {for}\,{t}<\tau\,\,\text{and}\,\,k_{prod} =(k_{deg}+k_{dil})\cdot\,e^{\beta\cdot(t-\tau)},\,for\,t\geq\tau\text{.}$$

We estimated the two parameters $\beta$ (decay rate), and $\tau$ (delay) by fitting the control condition (-dox) normalized to the 0 h timepoint through minimizing the sum of squared residuals using MATLAB’s fminsearch algorithm.

Using the estimated values for k_prod_, k_deg_ and k_dil_, we then estimated the two parameters describing the repression stability n and k_trans_, by fitting the model to the experimental data, as outlined in the following section.

Fluorescence intensities were normalized to scale between 0 and 1 using a min-max normalization approach, to enable direct comparison with ODE simulations, which describes the Dox-dependent reporter activity. For each treatment condition, the minimum fluorescence value F_min⁡​_ was defined as the lowest intensity measured in the dox-treated sample across all timepoints. The maximum F_max_​ was set as the fluorescence of the untreated control at 0 h. Normalized fluorescence at each timepoint t was computed as:

$$F_{norm}(t)=\frac{F_{treated(t)-F_{min}}}{F_{max-F_{min}}}$$

Protein levels and promoter ON state were initialized at 0, and the promoter OFF state at 1. To simulate immediate reactivation we set *n* = 0 and k_trans_=1000 (Fig. 6D + E, dotted line).

To optimize n and k_trans_, we tested model structures with *n* = 0–10 intermediate promoter states, where the average time to reactivation is given by $\Delta{t}=\frac{(n+1)}{k_{trans}}$.For each of these model structures, we simulated the protein kinetics for a range of k_trans_ values, and used profile likelihood to estimate k_trans_ together with a 95% confidence interval.

As the objective function we computed the normalized sum of squared residuals as, $X^{2}(k)=\sum(\frac{protein_{sim}-protein_{data}}{\sigma_{data}})^{2}$ estimated k as $\widehat{k}=argmin\left[X^{2}(k)\right]$, and chose the model structure that gave the lowest overall $X^{2}$.We derived the likelihood based 95% CI as the threshold where $\Delta{X^{2}}$ crosses the 95th percentile of the $X^{2}$ distribution with one degree of freedom $\left\{{{k\mid{X^{2}}(k)-X^{2}(\widehat{k})<\Delta_{0.95}=3.841}}\right\}$.The estimated parameters are provided in Additional file 5: Table S4. Notably, the data constrain the 95% CI bounds in 2i but not during differentiation, where only an upper bound is identifiable. 

As a control we performed a similar analysis for tdTomato deactivation upon dox withdrawal, where we would expect an immediate response. To estimate the differentiation-specific production rate, we used a control with continuous dox treatment (Additional file 2: Fig. S5H). The promoter was initially assumed to be in the ON state and then transitioned through n intermediate ON states to the OFF state.

### NGS data analysis

#### Nascent transcriptome assembly

To investigate antisense transcription genome-wide, we first generated a nascent transcriptome assembly based on TT-seq time course data set generated previously using the TXΔXicB6 mESC line [49, 50].

The data (GSE167356) was processed as described previously [49]. Additionally, blacklisted regions were removed from the BAM files using bedtools [intersect -v] (v2.29.2) [77]. Subsequently, we merged the BAM files of individual replicates and used deeptools (v3.5.1) to count reads genome-wide in 200 bp bins on the plus and minus strands separately with options [multiBamSummary bins --genomeChunkSize 2407883318 --centerReads -bs 200] [78]. Afterwards, the count tables were used as an input for the Genostan R package (2.24.0) to assign one out of 7 states to each bin depending on its transcription activity with options [initHMM(nStates = 7, method = “PoissonLogNormal”)] and [fitHMM(maxIters = 200)] [79]. Per strand, the top 5 states were assigned as transcribed bins. To detect putative TSSs, we identified bins who showed a >10-fold TT-seq signal difference between the previous and the consecutive two bins. For an independent detection of potential TSSs we used previously generated ATACseq data, as well as CUT&Tag data of H3K27ac, H3K4me3 and H3K4me1 (GSE167350 and GSE167353) in the same cellular context to assign promoter and enhancer states using ChromHMM (v1.19) [49, 54]. We then assigned each potential TSS detected in the TT-seq data as a true TSS only if the bin overlapped with a promoter/enhancer state from the ChromHMM analysis performed for the same time point, an annotated GENCODE TSS [53] or a CAGE peak recovered by the FANTOM5 consortium [55, 56]. The number of TSSs retrieved from either source is depicted in Additional file 2: Fig. S2A-C. To annotate the transcript associated with each verified TSS, the region was extended as far downstream as we could detect transcribed bins. Notably, we used the GENCODE annotation to fill holes between transcribed bins, if the TSS overlapped with an annotated GENCODE TSS. All other transcribed regions with an assigned TSS were removed from the analysis. To trim the annotated transcribed regions and increase the resolution, we counted reads in 10-bp bins covering the first and last 25% of each transcribed region using deeptools (v3.5.1) with options [multiBamSummary BED-file --centerReads] [78]. Then we detected the first bin from the start of the transcribed region that surpassed 1/4 of the mean read counts in the region and designated it as the exact TSS. Lastly, we detected the first bin from the end of the TU that surpassed 1/2 of the mean read counts in the region and designated it as the corrected TTS. TUs that overlapped on the same strand (intragenic TSS) were merged. A complete list of all TUs for days 0, 2 and 4 of differentiation is provided in Additional file 3: Table S2.

#### TU classification

Following transcriptome assembly, transcriptional units (TUs) were classified as either protein-coding or non-coding. Protein-coding TUs were defined as those with transcription start sites (TSSs) overlapping the promoter region of GENCODE-annotated protein-coding genes. Non-coding TUs were further categorized based on the genomic context of their TSSs. TUs with TSSs located within 1000 bp upstream of a protein-coding gene on the opposite strand were annotated as upstream antisense RNAs (uaRNAs). Those overlapping chromHMM-annotated enhancer regions were classified as enhancer RNAs (eRNAs). The remaining long non-coding RNAs (lncRNAs) were designated as either genic or intergenic, depending on whether their gene bodies overlapped with protein-coding TUs or not. The full annotation is provided in Additional file 3: Table S2.

#### Annotation of antisense overlaps

In order to find loci displaying antisense transcription, the generated (time-point-specific) annotations were used to find TU pairs on opposite strands that overlapped using GenomicRanges (v1.48.0) with options [findOverlaps ignore.strand = TRUE] [80]. Subsequently, the overlaps were classified as promoter overlap, 3’ overlap or intragenic overlap depending on the architecture of the two TUs. Expression of the entire TUs and within the overlaps was quantified using Rsubread (v2.10.5) with options [featureCounts(allowMultiOverlap = TRUE)] [81]. Data concerning the overlaps is supplied in Additional file 3: Table S2.

#### Gene ontology enrichment analysis

In order to identify biological pathways or molecular functions that are regulated by antisense transcription during the differentiation of mESCs, we performed gene ontology enrichment analysis using *ClusterProfiler* (v4.12.6) [82]. To this end, we first restricted the analysis to GENCODE-annotated transcripts that were detected in our nascent transcriptome assembly. Subsequently we generated gene sets associated with 3’ overlaps, promoter overlaps or no overlaps. Gene ontology enrichment was performed for each gene set with [enrichGO(OrgDb = org.Mm.eg.db, ont = “BP”, pAdjustMethod = “fdr”)]. Only terms with at least one condition with a FDR < 0.05 are shown in Additional file 2: Fig. S3A.

#### Annotation of composite overlaps

To create a composite set of overlaps throughout differentiation, we merged the assemblies of the three separate time points together. Afterwards, we once again annotated overlaps using GenomicRanges (v1.48.0) with options [findOverlaps(ignore.stand = TRUE)] [80]. Subsequently, we removed all overlaps shorter than 500 bp and counted reads within each overlap at the three timepoints within the TT-seq data using Rsubread (v2.10.5) with [featureCounts(allowMultiOverlap = TRUE)] [81]. We then calculated counts per million (CPM) for each condition and only kept overlaps with a minimum of three CPM at every time point on both strands combined. Lastly, we sorted the overlaps according to gene architecture as before and merged intersecting overlaps together.

#### Annotation of switching overlaps

In order to annotate overlaps which switched the dominant strand during differentiation, we first determined skewing in the composite overlap set at every time point and each of the 2 biological replicates, separately. To this end, we counted reads within the composite overlap set using Rsubread (v2.10.5) with [featureCounts(allowMultiOverlap = TRUE] and calculated CPM [81]. For each time point, we used a two-sided Student’s T-test with Benjamini-Hochberg correction to detect antisense pairs where transcription was significantly different between the two strands (FDR < = 0.1). Among those, we defined an overlap as skewed if the ratio between the two strands [CPM_plus/(CPM_plus + CPM_minus)] was below 0.35 (minus-biased) or above 0.65 (plus-biased). Overlaps were annotated as “switch” if they changed the direction of the bias between the plus and minus strand throughout differentiation. Overlaps that were biased at one time point and balanced at the other were annotated as “change”. Overlaps that showed the same bias at all time points or stayed balanced throughout were annotated as “no change”. The results were visualized as Sankey diagrams and/or scatter plots using tidyverse (v1.3.2) and ggsankey (v0.0.99999) [83]. A list of switch overlaps is given in Additional file 3: Table S2.

#### Generation of overlap controls

To study the effect of antisense transcription in 3’ or promoter overlaps in more detail we set out to generate an appropriate control set. To this end, we defined for each TU with a 3’ or promoter overlap an overlap-free region with similar length and expression. In detail, we filtered the day 0 or day 4 nascent transcriptome annotations for TUs longer than 2 kb with overlaps longer than 1 kb. Additionally, we removed every transcribed region with an overlap shorter than 5% or longer than 95% of the total transcript length. We then randomized the order of the overlap TUs and matched them to the free TUs in a loop, by matching on the maximal similarity in TPM and length. The free TU was then removed from the input, so that every TU could only be matched to an overlap TU once. We then created a “mock overlap” in each control matching the overlapped percentage of the partner TU. Lastly, the complete list of (mock) overlapping and free regions for promoter/3’ overlap transcription regions and controls were exported as BED files. Reads within the overlapping genes and the corresponding controls were quantified using Rsubread (v2.10.5) with [featureCounts(allowMultiOverlap = TRUE)] and expression throughout differentiation was quantified as TPM [81]. Significance was assessed using a ranked Wilcoxon sum test (p < = 0.05). A list of matched overlaps and control TUs are provided in Additional file 4: Table S3.

#### Generation of overlap lineplots

In order to investigate repression in 3’ overlaps, we compared expression along the 3’ overlap TUs and the corresponding, non-overlapping controls, to identify signs of premature termination due to polymerase collisions. To this end, we counted TT-seq reads using deepTools (v3.5.1) with [multiBamSummary scale-regions] in the overlapping and non-overlapping parts of the TUs separately [78]. In the controls, which had a similar length (see previous section), a region of the same length as the overlap in the corresponding 3’ overlap TU was designated as a mock-overlap. Expression was quantified in 12 bins in the free part and 8 bins in the (mock-)overlap part. The number of bins was chosen according to the mean percentage of overlap length in the 3’ overlap control set (42.1%). Additionally, we also quantified expression in the antisense direction as a control. Significance was assessed using a ranked Wilcoxon sum test (p < = 0.05).

#### Quantification of CUT&Tag data at promoters

The 3’ and promoter overlap sets detected at day 0, as well the corresponding control sets, were utilized to quantify H3K4me1, H3K4me3, H3K9me3, H3K27ac, H3K27me3, H3K36me3 and H2K119Aub data generated previously using CUT&Tag. The data (GSE167353) was processed as described previously [49]. Afterwards, counts were quantified from − 500 bp to + 200 bp around the TSSs using Rsubread (v2.10.5) with [featureCounts(allowMultiOverlap = TRUE)] [81]. The data was normalized as CPM and visualized as a heatmap depicting the log2 fold change between the mean counts in the 3’/promoter overlap sets and the respective controls. Significance was assessed using a ranked Wilcoxon sum test (p < = 0.01).

#### WGBS data processing

Adaptor as well as quality trimming of fastq files was performed using Trim Galore (v0.6.4) [84] with the options [--clip_R1 10 --three_prime_clip_R1 5 --clip_R2 15 --three_prime_clip_R2 5 --paired]. Subsequently, reads were aligned to the mouse genome (mm10) with BSMAPz [-q 20 -u -w 100] [85]. The resulting bam files were sorted and low quality mapped reads were removed using samtools [view -q 10] (v1.10) (Li 2009). Next, duplicate reads were removed with Picard (v2.7.1) using the options [MarkDuplicates REMOVE_DUPLICATES = TRUE]. Replicate bam files were merged and indexed using samtools. To obtain bedGraph files containing per-base CpG methylation metrics, MethylDackel (v0.3.0) was applied [extract --mergeContext --minDepth 2]. Mitochondrial regions were subsequently removed as well as blacklisted regions for mm10 using bedtools [intersect -v] (v2.29.2) [77, 86]. To obtain bigwig files for genome browser visualization, bedGraph files were converted using the UCSC software bedGraphToBigWig (v4) [87].

#### Quantification of WGBS data at promoters

In order to quantify the percentage of CpG-methylation in promoter regions, bedGraph files were loaded into Rstudio using the bsseq package (v1.32.0) [88]. Subsequently, CpG-methylation was calculated in promoters of 3’ and promoter overlapping TUs and controls analogous to the CUT&Tag analysis (−500 bp to + 200 bp around the TSSs) with options [getMeth(what = “perRegion”)]. Promoters without a called CpG were excluded from the analysis (< 0.01% of all promoters). The resulting percentages were then visualized using the tidyverse package (v1.3.2) [83] as a split violin plot.

### Experimental Methods

#### Cell lines

All experiments were performed in the female TXΔXic_B6_ line (clone A1) which is a F1 hybrid ESC line derived from a cross between the 57BL/6 (B6) and CAST/EiJ (Cast) mouse strains that carries a 773 kb deletion around the *Xist* locus on the B6 allele (chrX:103,182,701 − 103,955,531, mm10) and an rtTA insertion in the *Rosa26* locus [89]. The cell line was chosen, since it promotes Dox-inducible expression, but the Dox-inducible promoter present at the *Xist* gene in the parental line (TX1072) has been removed as part of the 773 kb deletion. Moreover, the TXΔXic_B6_ line had been used to generate a large data set in a previous study that was used in the genome-wide analyses.

#### mESC culture and differentiation

mESCs were grown on 0.1% gelatin-coated flasks in serum-containing medium supplemented with 2i and LIF (2iL) (DMEM (Sigma), 15% ESC-grade FBS (Gibco), 0.1 mM β-mercaptoethanol, 1000 U/ml leukemia inhibitory factor (LIF, Millipore), 3 µM Gsk3 inhibitor CT-99021, 1 µM MEK inhibitor PD0325901, Axon). Differentiation was induced by 2iL withdrawal in DMEM supplemented with 10% FBS and 0.1mM β-mercaptoethanol on fibronectin-coated (10 µg/ml) tissue culture plates.

#### Molecular cloning

The lentiviral vector carrying the landing pad (SP419) and the donor plasmid with the antisense locus were generated by standard molecular cloning techniques (details are given in Additional file 6: Table S5). The annotated plasmid sequences are provided as Supplemental file 1. Starting plasmids were kind gifts from Hana El-Samad (MTK0_057, MTK0_017) [90] and from Luca Giorgetti (#235_237_pUC19_Chr1_Ho_CuO_Cre_HyTK). Correct identity of all plasmids was confirmed using Sanger sequencing. A list of primers used for cloning is also provided in Additional file 6: Table S5.

#### Generation of the TxSynAS line carrying a synthetic antisense construct

To generate the landing pad cell line TX_LVLP (SC55), TXΔXic_B6_ mESCs were transduced sequentially with a lentiviral vector encoding an ERT2-Gal4 construct for inducible expression (SP265), and the landing pad construct (SP419) [90]. The ERT2-Gal4 is not used in this study.

##### Lentiviral transduction of the landing pad

For lentiviral transduction, 1*10^6^ HEK293T cells were seeded into one well of a 6-well plate and transfected the following day with the lentiviral packaging vectors: 1.2 µg pLP1, 0.6 µg pLP2 and 0.4 µg pVSVG (Thermo Fisher Scientific), together with 2 µg of the desired construct using Lipofectamine 2000 (Thermo Fisher Scientific). HEK293T supernatant containing the viral particles was harvested after 48 h. 0.2*10^6^ mESCs were seeded in a well of a 12-well plate in 2iL and transduced the next day with 1 ml of 5x concentrated (lenti-X, Clontech) and filtered viral supernatant with 8 ng/µl polybrene (Sigma Aldrich). Puromycin (1 ng/µl, Sigma Aldrich) or hygromycin (200 µg/ml, VWR) selection was started two days after transduction and kept until the selection control was dead but for at least 2 passages. To expand single clones, the cells were seeded at low densities in gelatin-coated 10 cm plates in 2iL medium and cultured until single colonies were visible (10 days). Then individual clones were picked and expanded. For the TX_LVLP line we selected clones that displayed high and homogenous expression upon BxB1-mediated integration of a GFP transcription unit (clones B1, A2).

##### BxB1-mediated integration

The TxSynAS line was then generated from the TX_LVLP line (clone B1) by insertion of the synthetic antisense locus into the landing pad through BxB1-mediated integration. To this end, the antisense plasmid (SP505) and a BxB1-encoding plasmid (SP225, Addgene #51271) were transfected with Lipofectamine 3000 (Thermo Fisher Scientific) in a 3-to-1 target-to-integrase ratio. 48 h post transfection, blasticidin selection (5 ng/µl, Roth) was started, and single clones were expanded while selection was maintained. Clone C6 was chosen for all further experiments since it displayed high and homogenous GFP expression in the absence of doxycycline.

##### Constraints and design considerations for the synthetic antisense locus

We initially tried to encode a reporter on each strand of the DNA sequence between two convergent promoters. We tested multiple different arrangements, with the antisense reporter encoded either in the 3’ UTR or 5’ UTR of the sense strand. We were, however, unable to find a design, where reporters from both strands could be detected by flow cytometry, maybe because the insertion of the antisense reporter sequence destabilized the transcripts. In addition, we were unable to further increase transcription from the constitutively active promoters through combination with an inducible promoter, which would not allow us to switch the locus’ expression state. To circumvent these limitations, we implemented a unidirectional design, in which the sense gene (GFP) is driven by a constitutive EF1α promoter. To control transcription in the antisense direction, we utilized a commonly used Doxycycline-inducible bidirectional promoter (pTRE-Tight) which drives antisense transcription across the GFP TU, and a tdTomato TU in the sense direction. GFP and tdTomato were fused to a conditional FKBP destabilizing domain to accelerate protein turnover and thus temporal resolution of our measurements. However, the fluorescent signal of the destabilized reporters was too weak for reliable detection by flow cytometry. Therefore all experiments were performed in the presence of Shield1, which stabilizes the FKBP domain.

#### Memory experiment

For experiments, where reporter fluorescence was quantified by flow cytometry, all media were supplemented with 1µM Shield1 ligand (Takara, #632189) to stabilize both reporters, because the signals were too weak in the destabilized state without Shield1. 0.3*10^6^ cells, grown in 2iL, were seeded in a well of a 6-well plate in media containing 0 µg/ml or 2 µg/ml doxycycline, either in undifferentiated state (2iL, gelatine-coated culture dish) or with inducing differentiation (−2iL, fibronectin-coated culture dish). After two days, cells were harvested and seeded at a density of 7500 cells per well in a 96-well plate into media without Dox, except for controls that were maintained in 2 µg/ml Dox, and assayed by flow cytometry 0 h, 8 h, 1 d, 2 d, 3 d, and 4 d later. To estimate background from autofluorescence a negative control (landing pad cell line w/o antisense construct integration) was measured in parallel in 2iL or −2iL.

#### Flow cytometry

Cells were analyzed using the BD FACSCelesta flow cytometer (Beckton Dickinson, IC-Nr.: 68186, Serial-Nr.:R66034500035) with 2-Blue6-Violet4-561YG laser configuration, and the following filter settings: 530/30 for GFP, 586/15 for tdTomato and 450/40 for TagBFP. The instrument was also equipped with a BD High Throughput Sampler. The sideward and forward scatter areas were used for live cell gating. The height and width of the forward scatter were used for singlet/doublet differentiation. At least 10,000 events were recorded per replicate, initial condition, and doxycycline concentration. Fcs files were gated using RStudio with the *flowCore* (v1.52.1) and *openCyto* packages (v1.24.0) [91, 92]. The mean fluorescence intensity (MFI) of the singlets was calculated. Background correction was performed by subtracting the MFI of the TX_LVLP line in the respective media.

#### WGBS

TXΔXic_B6_ were differentiated for 0, 2 or 4 days and genomic DNA was extracted using the DNeasy Blood and Tissue Kit (Qiagen). Bisulfite converted DNA libraries were prepared using the Accel-NGS Methyl-Seq DNA library kit (SWIFT BIOSCIENCES). In brief, 200ng (in 50 µl lowTE) of purified DNA were fragmented to ~ 350 bp using the Covaris S2 system (10% duty cycle, intensity 5 for 2 × 45 s in Covaris AFA tubes) followed by a concentration step with Zymo columns (DNA Clean & Concentrator). 20 µl of DNA were used for bisulfite conversion over night with the Zymo EZ-DNA methylation Gold Kit.

Bisulfite converted DNA was fully denatured by 2 min incubation at 95 °C and immediately transferred to ice. To anneal a truncated adapter, 15 µl of denatured DNA was mixed with 25 µl of Adaptase reaction mix (SWIFT) and incubated for 15 min at 37 °C, 2 min at 95 °C, and then cooled to 4 °C. Extension and second strand synthesis by using a primer complementary to the truncated adapter was performed mixing the Adaptase reaction mix with 44 µl of extension reaction mix (SWIFT) and incubation at 98 °C for 1 min, 62 °C for 2 min, 65 °C for 5 min and cooling to 4 °C. The samples were cleaned with 1.2 volumes of Ampure XP beads, 80% ethanol and eluted in 15 µl. Ligation of the second (truncated) adapter was performed at 25 °C for 15 min followed by an additional bead clean up with 1 volume of AmpureXP beads and 80% ethanol. Samples were individually indexed using unique dual indexed primer sets with 6 cycles of a slightly modified PCR program (30s @ 98 °C, 6 cycles of 15 s @ 98 °C, 30 s @ 60 °C, 60 s @ 68 °C, followed by a final 5 min incubation step at 68 °C. Libraries were finally cleaned up with 1 volume of Ampure XP beads. Quality was assessed using Agilent’s Bioanalyzer and concentration was determined by qPCR. Libraries were pooled equimolarly and sequenced on a NovaSeq 6000 S2 flowcell (Illumina) in paired end 150 mode to yield ~ 120-278mio fragments [93–101].

## Supplementary Information

Additional file 1: Table S1. Simulations of antisense transcription: parameter ranges tested and statistics of FST analysis.

Additional file 2: Supplementary figures.

Additional file 3: Table S2. Genome-wide annotation of antisense transcription: De novo annotation of transcribed regions; detected antisense loci; transcriptional activity of all transcribed regions, and of antisense loci; composite antisense loci; switching antisense loci (related to Fig. 4).

Additional file 4: Table S3. Characterization of antisense loci: Control regions used for comparison; statistical analyses comparing overlapping, and non-overlapping control regions (related to Fig. 5).

Additional file 5: Table S4. Summary of best fit parameter values for the ODE model that was used to quantify repression stability, along with 95% confidence intervals where available (related to Fig. 6).

Additional file 6: Table S5. Molecular Cloning: cloning strategies plasmids and primers.

Additional file 7: Software. Summary of software used in this study.

Supplementary Material 8. Annotated sequences of plasmids used in the study (SP505, SP419) in Genebank format.

## Data Availability

Code used in the current study is available at Github [102], (https:/github.com/EddaSchulz/Antisense_paper) under the MIT license. A version of the source code used in this manuscript has been archived in Zenodo [103], (10.5281/zenodo.17481422). DNA methylation data [104], generated in this study is available on GEO under accession number GSE253792.
