# Supplementary material for: Antisense transcription can induce expression memory via stable promoter repression
Source: Genome Biol. 2025 Dec 20;26:430. doi: 10.1186/s13059-025-03875-1 (PMC12720443; doi:10.1186/s13059-025-03875-1)
Supplement: Supplementary file 2 — Additional file 2: Supplementary figures. [file 13059_2025_3875_MOESM2_ESM.pdf]

Additional File 2

Supplementary Figures

Antisense transcription can induce expression memory via stable promoter repression

Verena Mutzel[1], Till Schwämmle[1], Svearike Oeverdieck[1], Lucija Librenjak[1], Benedikt Boesen[1], Melissa Bothe[1], Rutger AF Gjaltema[1], Ilona Dunkel[1], Gemma Noviello[1], Edda G Schulz[1]

[1] Systems Epigenetics, Otto Warburg Laboratories, Max Planck Institute for Molecular Genetics, 14195 Berlin, Germany

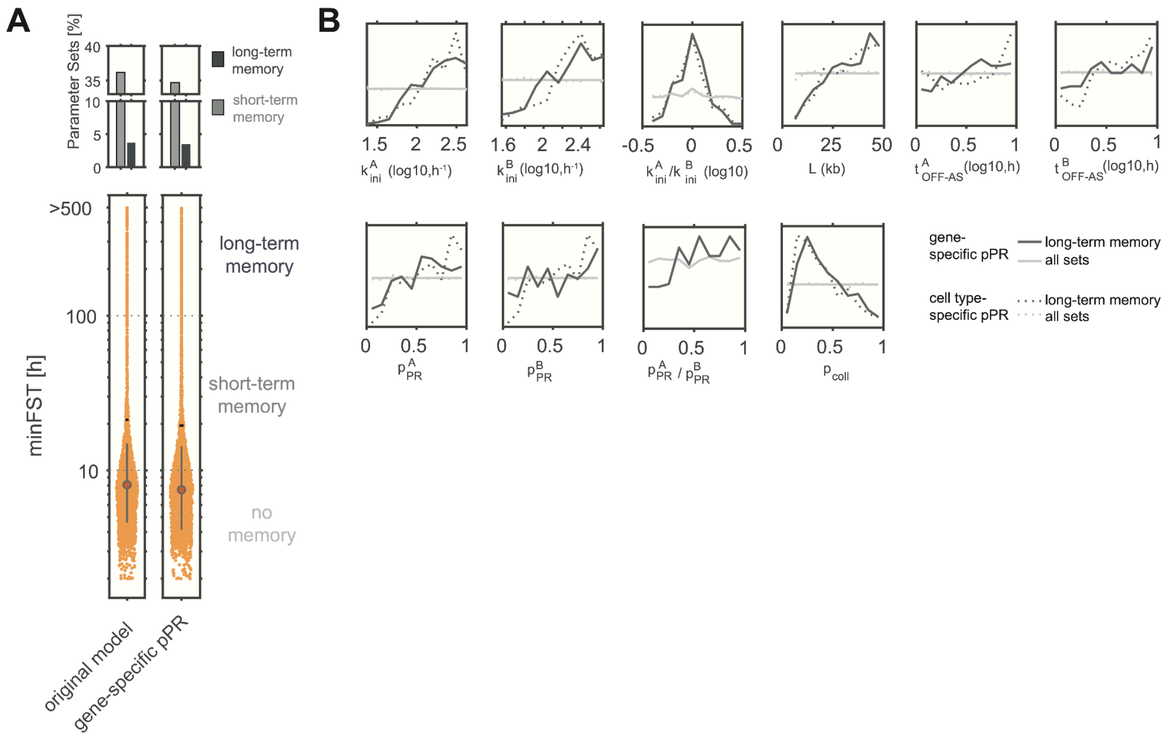

Fig. S1: pPR as a gene-specific parameter.

Related to figure 2

A) Simulation of 7,000 randomly sampled parameter sets with mutual promoter overlap, with pPR as a cell type- (left, same as Fig. 2C) or gene- specific (right) parameter. Distribution of minFST (bottom) together with percentage of parameter sets displaying short-term (gray) and long-term (dark gray) expression memory (top).

B) Distribution of parameter values when pPR is a gene-specific (solid) or a cell-type specific (dotted) parameter, across all parameter sets (light gray), and those displaying long-term expression memory (dark gray).

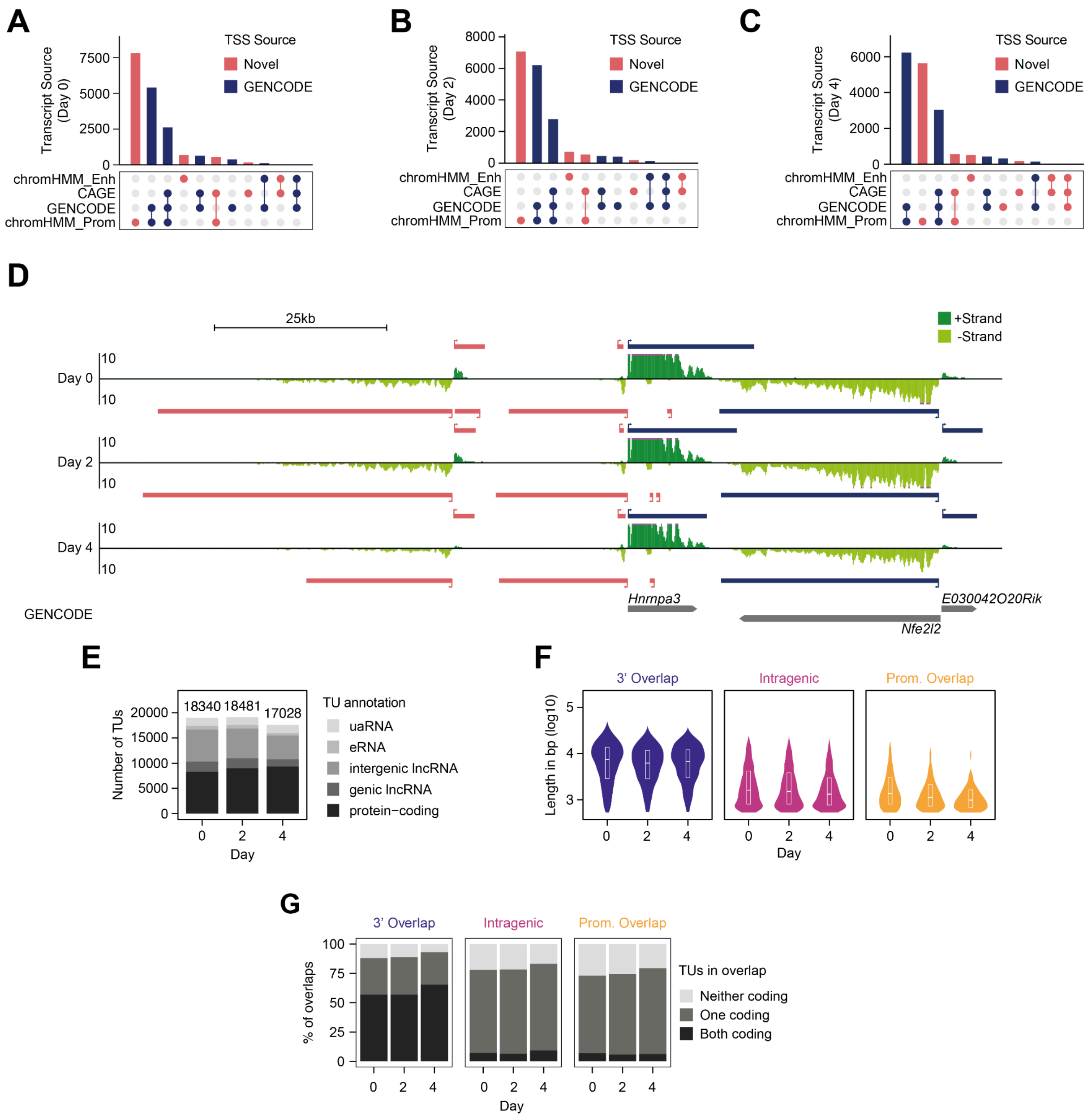

Fig. S2. Genome-wide annotation of antisense transcription

Related to figure 4.

A-C) UpSetR plots showing the sources for the TSSs of annotated TUs for day 0 (A), 2 (B) and 4 (C) of differentiation. TSSs with GENCODE annotations are shown in blue. Novel TSS are shown in red.

D) Genome browser screenshot of an example locus with nascent transcriptome assembly. TUs on the plus strand are shown above and TUs on the minus strand below the tracks for each timepoint. Novel TUs are colored in red, while GENCODE transcripts are shown in blue. TT-seq signal that extends beyond the scale is marked with purple on the edge of the tracks.

E) Number of annotated transcribed regions per time point. TUs are separated by annotation into different gene types including upstream antisense RNA (uaRNA), enhancer RNA (eRNA), intragenic long non-coding RNA (lncRNA), genic lncRNA and protein-coding.

F) Length of identified overlaps separated by type at days 0, 2 and 4 of differentiation.

G) Percentage of overlap pairs with no, one or two protein-coding genes, separated by overlap type and time point of differentiation.

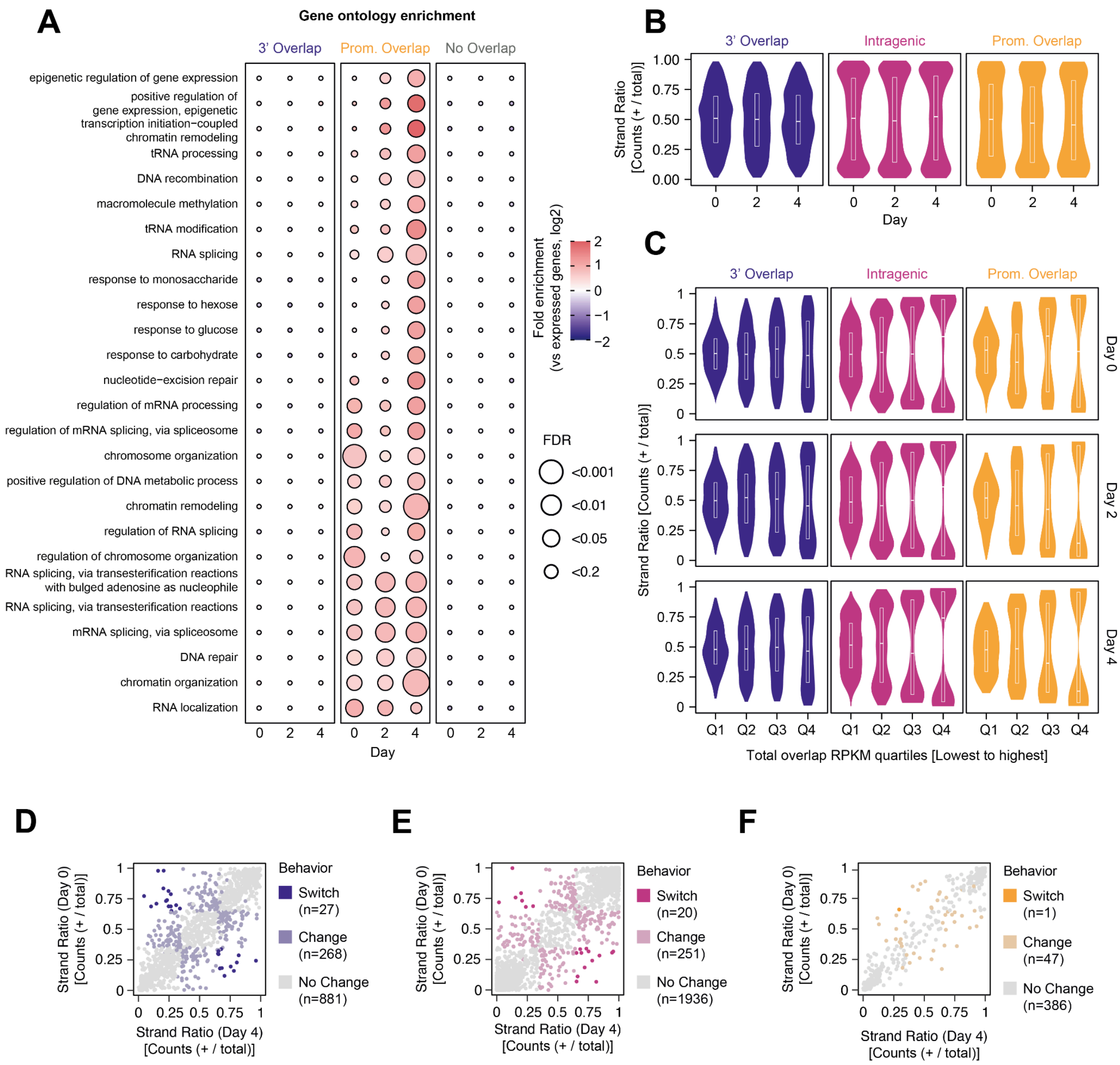

Fig. S3. Functional description of overlap loci

Related to figure 4.

A) Gene ontology analysis was performed for GENCODE-annotated genes involved in 3' and promoter overlaps against a background set of expressed genes (detected by the nascent transcriptome annotation). Genes without any overlaps were used as a control. All gene ontology terms with an FDR<0.05 in at least one condition were included in the figure.

B) Strand ratio within the different assemblies separated by day and overlap type. The ratio was calculated as the fraction of counts mapping to the plus strand.

C) As in (B), but separated into quartiles by total overlap expression. Overlap expression was quantified as reads per kilobase million (RPKM) across both strands in the overlapping regions.

(D-F) Scatter plot comparing the strand ratio of 3' overlaps (G), intragenic overlaps (H) and promoter overlaps (I) at day 0 and day 2. An overlapping region was categorized as biased ('change'), if transcription was significantly different between the plus and minus strands (FDR<=0.1, Student's T-test with Benjamini-Hochberg correction) and the strand ratio was >= 0.65 (plus bias) or <= 0.35 (minus bias). An overlap was annotated as 'switch', if it changed from one bias to the other and as 'change' if it changed from no bias to bias (or vice-versa).

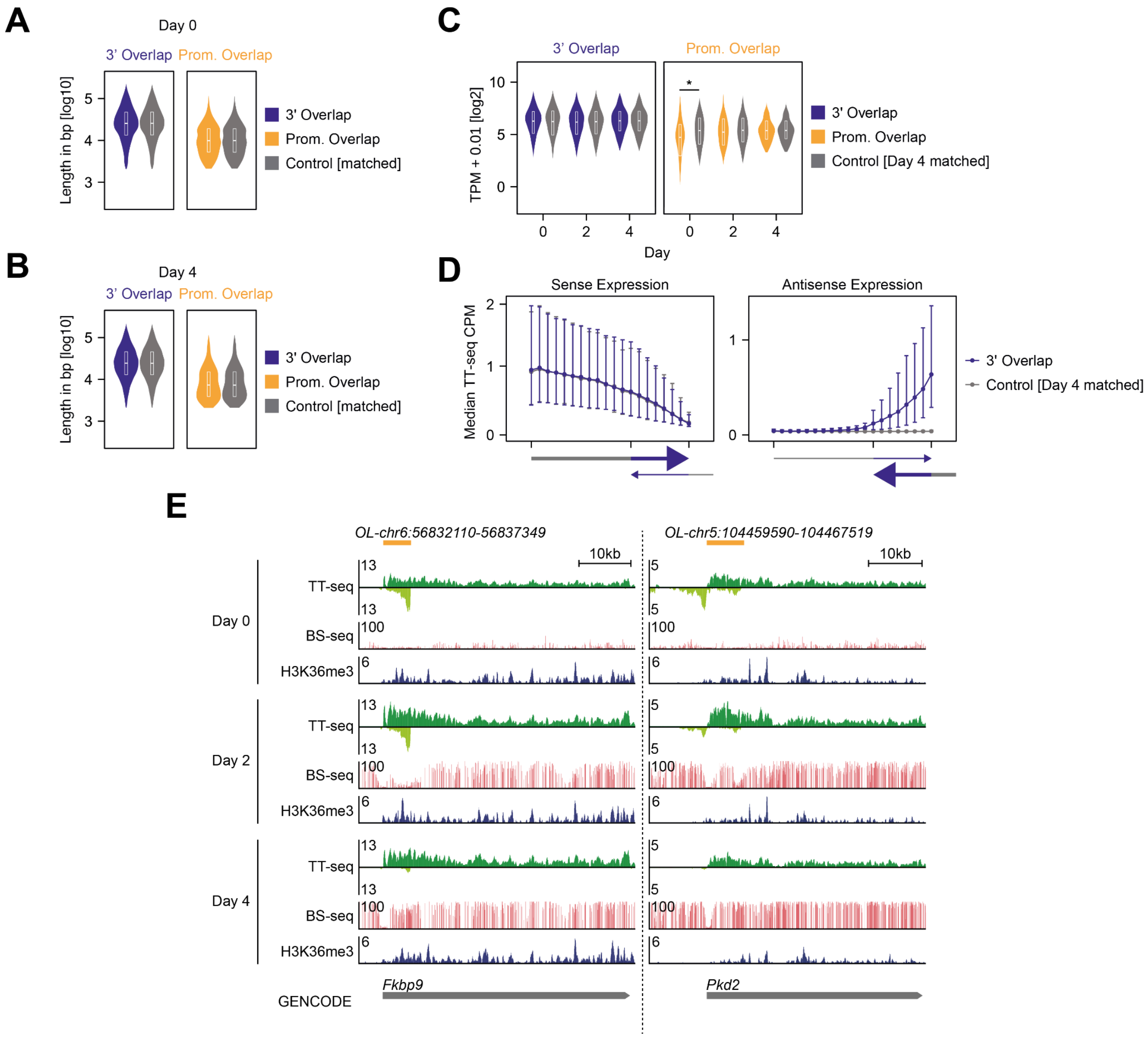

Fig. S4. Systematic comparison of antisense loci with overlap-free genes

Related to figure 5.

A-B) Length comparison between 3'/promoter-overlapping TUs and matched controls at day 0 (A) or 4 (B).

C) Expression measured by TT-seq of 3' and promoter-overlapping transcribed regions, annotated based on nascent transcription at day 4 of differentiation in comparison to matched controls. Significance was assessed using a ranked Wilcoxon sum test (p<=0.01).

D) Binned line plot showing sense or antisense expression of TT-seq data in 3' overlap transcribed regions and controls at day 4. Expression was quantified in 12 "free" bins and 8 "overlapping" bins. The big dots depict the median of all transcribed regions, while the upper and lower whiskers depict 3rd and 1st quartiles respectively. Significance was assessed using a ranked Wilcoxon sum test (p<=0.01).

E) Genome browser screenshot showing TT-seq, BS-seq and H3K36me3 CUT&Tag data for two promoter overlaps at which transcription correlates with DNA-methylation. TT-seq reads from the plus-strand are colored in dark green, while reads from the minus-strand are shown in light green. The location of the overlaps is shown above the tracks

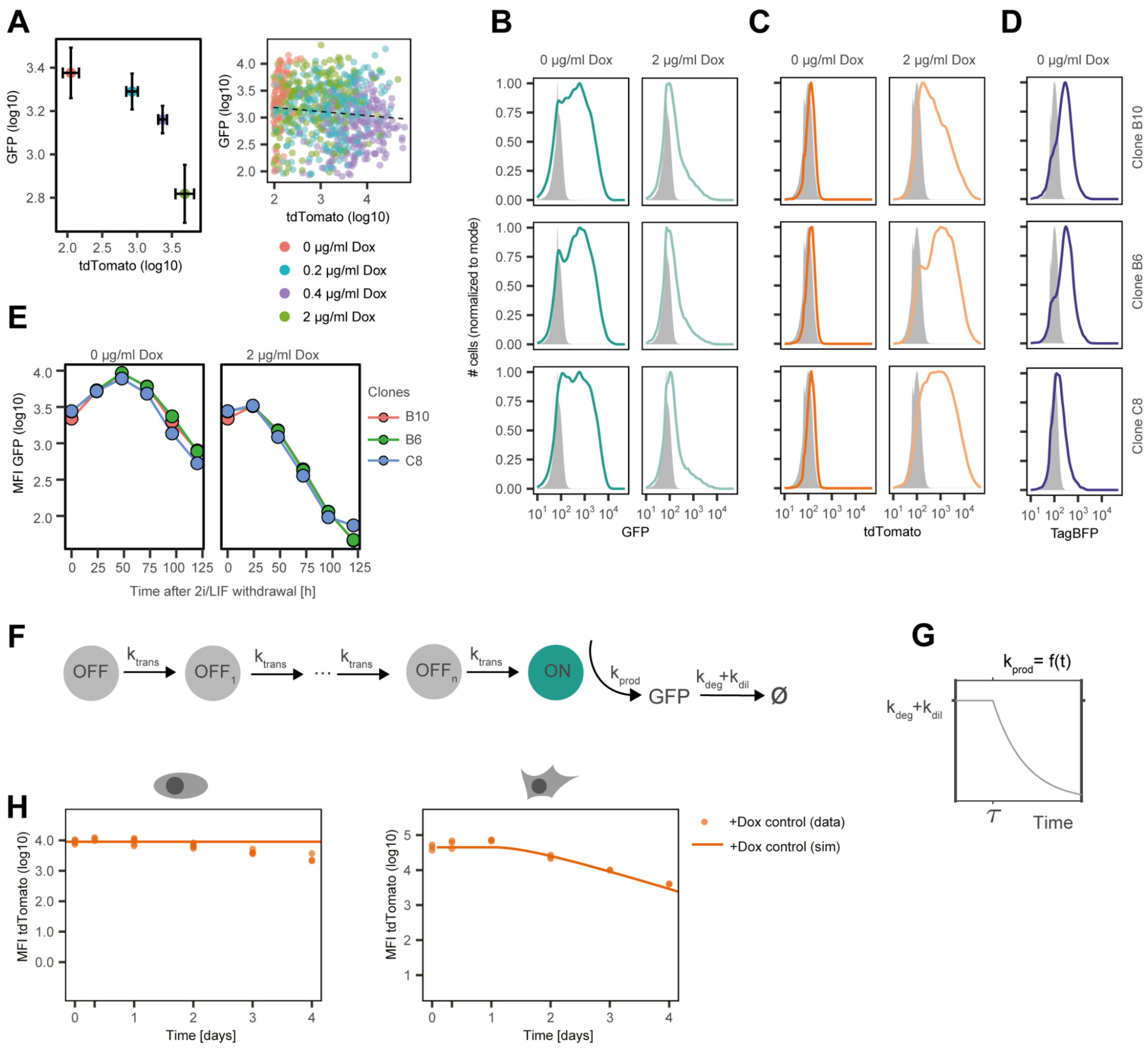

Fig. S5: Expression kinetics during differentiation in TxSynAS cells together with ODE model of reporter kinetics to quantify memory

Related to figure 6

A) Left: Anticorrelation of mean GFP and tdTomato across four different Dox concentrations. Dots show the mean, error bars indicate the s.d. of n = 4 measurements. Right: Scatter of GFP vs tdTomato fluorescence signal in single cells (subsampled to n=1000 cells above MFI of negative control). The negative correlation in single cells is weakened by confounding factors such as cell size, global transcriptional activity, measurement noise, and unequal exposure to Shield.

B-D) GFP (B), tdTomato (C) and TagBFP (D) expression in three additional clones of the TxSynAS line, where the synthetic locus was integrated in two different genomic positions, cultured for 2 days in the presence (light colors) or absence (dark colors) of Dox. TagBFP is expressed only upon integration into the landing pad.

E) GFP expression upon differentiation by 2i/LIF withdrawal in single clones of the TxSynAS line with different genomic antisense integration sites in the presence of 0µg/ml (left) and 2µg/ml (right) doxycycline.

F) Structure of the ODE model used to quantify the stability of antisense-induced repression. The GFP promoter starts in the OFF state, and transitions through n intermediate states (OFF_1-OFF_n), before reaching the transcriptionally active ON state, from which GFP protein is produced, that then is

degraded. The number of intermediate OFF states n and the transition rate k_{trans} were fitted to the experimental data (see methods).

G) Differentiation dependence of the GFP production rate estimated based on control data (-dox).

H) Background-corrected mean fluorescence intensity (MFI) of tdTomato under continuous Dox treatment in mESC medium FBS/2i/LIF (left) and upon differentiation by 2i/LIF withdrawal (right, used to estimate differentiation specific production rate). Lines represent simulation of the ODE model (see Suppl. Table 4).
